# Topological light-trapping on a dislocation

**DOI:** 10.1038/s41467-018-04861-x

**Published:** 2018-06-25

**Authors:** Fei-Fei Li, Hai-Xiao Wang, Zhan Xiong, Qun Lou, Ping Chen, Rui-Xin Wu, Yin Poo, Jian-Hua Jiang, Sajeev John

**Affiliations:** 1 School of Electronic Science and Engineering, Nanjing University, Nanjing, 210093 China; 2 College of Physics, Optoelectronics and Energy, & Collaborative Innovation Center of Suzhou Nano Science and Technology, Soochow University, 1 Shizi Street, Suzhou, 215006 China; 3 Department of Physics, University of Toronto, 60 Saint George Street, Toronto, ON M5S 1A7 Canada

## Abstract

Topological insulators have unconventional gapless edge states where disorder-induced back-scattering is suppressed. In photonics, such edge states lead to unidirectional waveguides which are useful for integrated photonic circuitry. Cavity modes, another type of fundamental component in photonic chips, however, are not protected by band topology because of their lower dimensions. Here we demonstrate that concurrent wavevector space and real-space topology, dubbed as dual-topology, can lead to light-trapping in lower dimensions. The resultant photonic-bound state emerges as a Jackiw–Rebbi soliton mode localized on a dislocation in a two-dimensional photonic crystal, as proposed theoretically and discovered experimentally. Such a strongly confined cavity mode is found to be robust against perturbations. Our study unveils a mechanism for topological light-trapping in lower dimensions, which is invaluable for fundamental physics and various applications in photonics.

## Abstract (web summary)

Although topological confinement of waves to the edges is common, lower-dimensional wave confinement is scarce. Here, Li et al. demonstrate that concurrent wavevector and real-space topology can lead to a topologically protected zero-dimensional cavity mode in a two-dimensional photonic crystal.

## Introduction

Photonic crystals (PhCs) are periodic structures of electromagnetic materials which offer versatile tailoring of photonic spectrum and wave dynamics^1–3^. Recently, photonic quantum anomalous Hall effects^4–11^, photonic Floquet topological insulators^12–14^, photonic quantum spin Hall insulators^15–22^, topological photonic quasicrystals^23–26^, and photonic Zak phases^27–29^ are realized or proposed using various PhCs. Topology^30–36^ is revealed as a mechanism for light-trapping on the edges of PhCs^4–29^, leading to topological surface states which are much more robust than conventional surface states^3^. However, until now, topological light-trapping at (sub-)wavelength scale is achieved only for edges which have one less dimension than the bulk. Lower-dimensional light-trapping protected by topological mechanism has not yet been discovered in photonics. In two-dimensional (2D) photonic systems, such lower-dimensional wave localization, once realized, can lead to robust cavity modes (zero-dimensional (0D) photonic states), which are demanded in photonics and hybrid quantum systems^37^.

Here, we predict theoretically and observe experimentally robust light-trapping into a 0D cavity mode in a 2D PhC, as induced and protected by the dual-topology mechanism. The characteristic localization length *l*_loc_ of the cavity mode is close to the wavelength in vacuum *λ*. The frequency of the topological cavity mode is found to be more robust than conventional PhC cavities. Strong and robust light-trapping is ubiquitously useful in the state-of-art photonics such as miniature photonic devices, integrated photonic/quantum chips, and cavity quantum electrodynamics. We emphasize that although the dual-topology mechanism was first proposed in electronic systems^38–40^, it is much more feasible to observe and utilize dual-topology to induce wave localization in photonics than in electronics. In fact, observation of such *d*-2 dimensional wave localization in a *d*-dimensional electronic system has not yet been achieved.

## Results

### Dual-topology mechanism

**Fig. 1:**
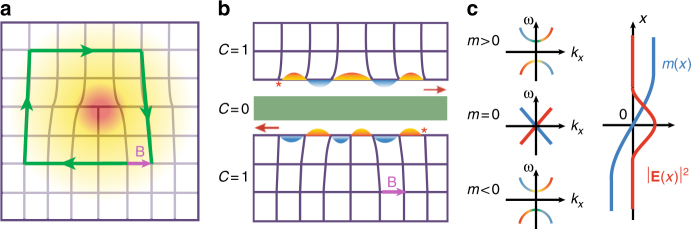
Light-trapping on a dislocation due to dual-topology. **a** Schematic of a dislocation and the Burgers vector in a rectangular lattice. A photonic-bound state (red region) is localized at the center of the dislocation. **b** Illustration of the cut-and-glue technique. Inserting a chunk of photonic crystal with trivial topology (the green strip) into a topological PhC with a dislocation yields two edge channels at opposite boundaries. Because of the additional lattice period in the lower edge, on the left (right) side of the dislocation there is a *π* (no) phase difference between the two edge states. **c** The *π* phase shift in the edge channels yields a sign-change in the Dirac mass across the dislocation, forming a Dirac mass domain-wall which results in a photonic Jackiw–Rebbi soliton mode

The proposed structure is a rectangular-lattice PhC with spatial periodicities *a*_*x*_ and *a*_*y*_ along the *x* and *y* directions, respectively. The photonic band gap (PBG) has a Chern number ${\it{{\cal C}}} = 1$ and the Zak phase^33^ along the Brillouin zone (BZ) boundary line XM is *θ*_XM_ = *π*. These two special properties constitute the nontrivial topology in wavevector space. On the other hand, a dislocation is an object with real-space topology: any close-loop of lattice translation including the dislocation has a mismatch between the starting and ending points. The vector connecting these points, the Burgers vector, serves as the real-space topological invariant (see Fig. 1a).

To elucidate the light-trapping mechanism through the dual-topology, we employ the cut-and-glue technique^38^ (see Figs. 1b and c) which consists of two steps: first, a chunk of PhC with trivial topology is inserted into the dislocation structure which is then split into two halves. This step introduces two one-way edge channels at the opposite boundaries of the chunk, due to the wavevector space topology. The dispersions of these two edge channels must intersect at a time-reversal invariant wavevector. The nontrivial Zak phase along the XM line ensures such an intersection to be at $K_x = \frac{\pi }{{a_x}}$ ^27,38^. For a finite-width chunk, the tunnel coupling between the opposite edges opens a gap at the intersection (Dirac) point. These two coupled edges can be described by the 1D massive Dirac Hamiltonian, ${\it{{\cal H}}} = vq_x\sigma _z + m\sigma _x$, where *v* is the group velocity of the edge states, *σ*_*z*_ = ±1 represents the two edge channels and *q*_*x*_ ≡ *k*_*x*_ − *K*_*x*_ is the wavevector relative to the Dirac point. The Dirac mass *m* (*m* is real, see Supplementary Note 1) depends on the inter-edge coupling. In the weak coupling regime, the Dirac mass is determined by the overlapping integral of the electromagnetic fields of the two edge states^3^, i.e., $m \propto {\int} {\mathrm{d}\,{\mathbf{r}}\,\left( {{\mathbf{E}}_ + \cdot \widehat \varepsilon \cdot {\mathbf{E}}_ - ^ \ast + {\mathbf{H}}_ + \cdot \widehat \mu \cdot {\mathbf{H}}_ - ^ \ast + {\mathrm{c}}.{\mathrm{c}}.} \right)}$ where the subscripts ± represent the edge states at the upper and lower boundaries, respectively. Due to the dislocation, the Dirac mass becomes position dependent, since the upper and lower edges experience different numbers of lattice translations (see Fig. 1b). If the Burgers vector is **B** = (*a*_*x*_, 0), the phase difference between the two edge states will experience a *π*-phase elapse across the dislocation, since *K*_*x*_*a*_*x*_ = *π*. This *π*-phase elapse leads to a sign-change in the Dirac mass, forming a mass domain-wall at the dislocation. According to the Jackiw–Rebbi theory^30^, there will be a photonic-bound state localized at the dislocation center.

The second step in the cut-and-glue procedure is to glue the two halves together by reducing the width of the chunk gradually to zero. As the width of the chunk reduces, the inter-edge coupling becomes stronger and stronger. The magnitude of the Dirac mass increases and the edge states are gradually moved into the bulk bands. However, because the Dirac mass domain-wall persists as it is guaranteed by the real- and wavevector-space topology, the Jackiw–Rebbi soliton mode always exists in the PBG, even when the chunk of topologically trivial PhC is removed.

The above scenario of light-trapping by the dual-topology mechanism can be mathematically summarized as^38–42^

1 $$N_{{\mathrm{loc}}} = \frac{1}{\pi }{\mathbf{B}} \cdot {\mathbf{\Theta }}:{\mathrm{mod}}\,2,$$

where ${\mathbf{\Theta }} = \left( {\frac{{\theta _{{\mathrm{XM}}}}}{{a_x}},\frac{{\theta _{{\mathrm{YM}}}}}{{a_y}}} \right)$ with *θ*_XM_ and *θ*_YM_ being the Zak phases along the XM and YM lines with contributions from all bands below the gap, respectively, and *N*_loc_ = 0 or 1 is the number of localized photonic modes. The existence of the mass domain-wall and the soliton mode has a *Z*_2_ nature, reflecting whether the Dirac mass switches sign or not (see Supplementary Note 2). We emphasize that the Chern number, though not appeared in the above equation, provides the ground for the edge states and the Jackiw–Rebbi soliton^41–44^. This crucial requirement differs our mechanism from those in refs. ^45,46^, beside the strong wave localization observed in this work.

### Design and characterization of the PhC

**Fig. 2:**
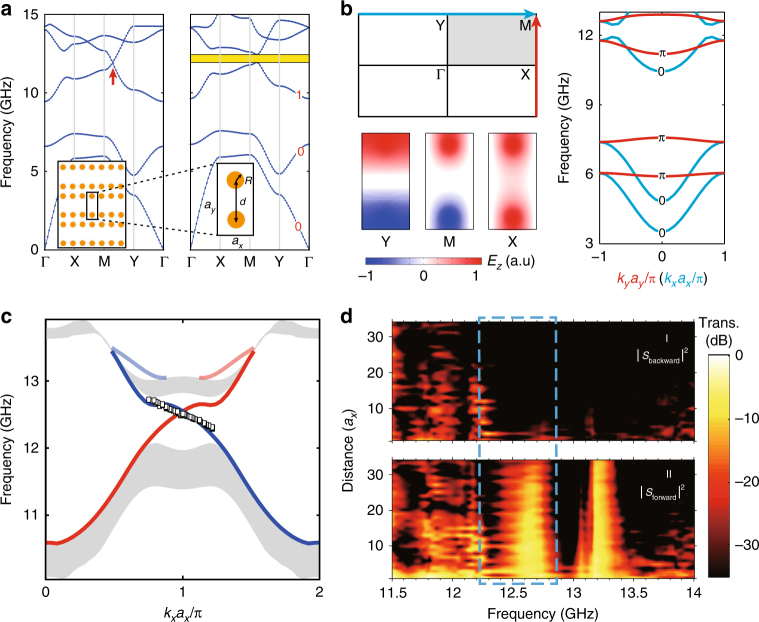
Topological photonic crystal. **a** Design of the photonic crystal and photonic band gap with nontrivial topology (Chern numbers labeled by red numbers) by gapping out the Dirac points (red arrow) using an external magnetic field. **b** Illustration of the Brillouin zone, the dispersion, and the Zak phase along the XM (YM) line (the red (cyan) curves), and the field profiles of the first band. **c** Edge states from simulation (red and blue curves) and experiments (square blocks, only at one edge). Bulk bands are represented by gray areas. **d** Nonreciprocal light propagation: the forward (panel I) and backward (panel II) transmission as functions of frequency and the distance between the source and the detection points along the edge. The black dashed box indicates pronounced nonreciprocal photon transport along the edge

To realize light-trapping due to the dual-topology mechanism, we design a rectangular-lattice PhC with two yttrium iron garnet (YIG) cylindrical pillars in each unit cell. With the metallic cladding above and below, the bulk photonic bands of interest here are the 2D transverse-magnetic (TM) harmonic modes. Magnetized by a magnetic field of 900 Oe along the *z* direction, a PBG (denoted as PBG II) between the third and fourth bands with Chern number ${\it{{\cal C}}} = 1$ is developed for *a*_*y*_ = 2*a*_*x*_ = 24 mm, *R* = 2 mm, and *d* = 17 mm. This topological PBG is realized by the gyromagnetic effect which gaps out the two Dirac points (indicated by the red arrow) on the YM line (see Fig. 2a). The dispersions along the BZ boundary lines YM and XM after the gap opening are shown in Fig. 2b together with the Zak phases along these lines. The Zak phases and the Chern number are calculated numerically using the Wilson-loop approach (see Supplementary Note 3). The electric field profiles at high symmetry points (Fig. 2b) indicate connection and consistency between our diatomic photonic unit cell and the Su–Schrieffer–Heeger model^31^: the parity inversion between the X and M points leads to *θ*_XM_ = *π*, while the absence of parity inversion between the Y and M points gives *θ*_YM_ = 0^27,33^. We obtain from numerical calculations that the Zak phases are *θ*_YM_ = 0 and *θ*_XM_ = *π* for the first three bands (Fig. 2b).

The edge states at opposite boundaries indeed intersect at $K_x = \frac{\pi }{{a_x}}$, as verified by the finite-element simulation method (see Fig. 2c), which is consistent with the nontrivial Zak phase along the XM line, *θ*_XM_ = *π* ^27,38^. The edge states dispersion measured in experiments using the Fourier-transformed field scan method (see Methods) agrees well with the dispersion from the finite-element simulation, as shown in Fig. 2c. The nonreciprocal photon flow along the edge channel, characterized by the difference between the forward and backward transmission, is presented in Fig. 2d. Pronounced nonreciprocal photon flow exists in the frequency window of 12.21–12.84 GHz (indicated by the black dashed box in Fig. 2d), while relatively weak nonreciprocal photon transport exists for lower frequencies as well. The bulk band gap is between 12.05 and 12.60 GHz (see Supplementary Note 4). The edge states spectrum spans a much larger frequency range of about 10.7–13.2 GHz. Pronounced nonreciprocal transmission may appear only in a fraction of this range, which is possibly caused by the impedance mismatch between the feed probe and the edge/bulk states for the finite-size sample studied in our experiments. A higher frequency window of nonreciprocal transmission is also visible, which is likely due to the nonreciprocal edge states in the higher PBG (the shallow red and blue curves in Fig. 2c) and irrelevant to the study in this work.

### Photonic realization of the dual-topology mechanism

**Fig. 3:**
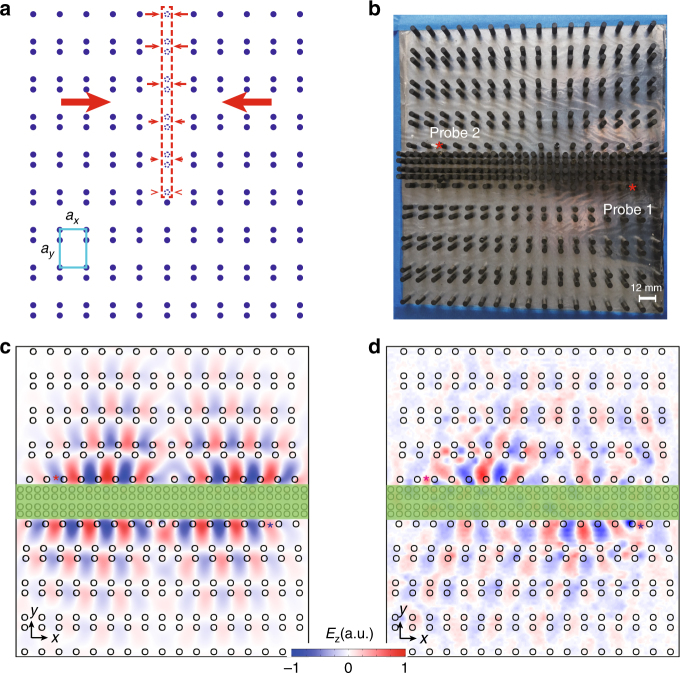
Dislocation in the topological photonic crystal. **a** Forming the dislocation. A half column of unit cells in the red-dashed region is taken away. The rest of the photonic crystal is compressed toward the red-dashed region to restore the lattice order except at the dislocation. **b** Experimental sample with a dislocation of Burgers vector **B** = (*a*_*x*_, 0) and an inserted chunk of topologically trivial photonic crystal. **c** Confirmation of the *π* phase elapse of the relative phase between the opposite edge channels in the presence of the dislocation from simulation. **d** Field distribution measured in the two-points pumping scheme

In our design, the dislocation is formed by first taking half column of unit cells (indicated by the red-dashed rectangular region in Fig. 3a) away from the perfect PhC. We then compress the rest of the PhC to the vacancy left to restore the lattice order. This can be done by continuous deformation of the whole PhC, leaving only the dislocation center as a structure defect. The final appearance of the sample measured in experiments (including an inserted chunk of PhC with trivial band topology) is given in Fig. 3b. The topologically trivial PhC is designed using the same YIG pillars but with different lattice constants $\left( {a_x \!\! \prime = a_x/2} \right),\left( {a_y \!\! \prime = a_y/2} \right)$ and the distance between the pillars (*d*′ = 5 mm) (see Supplementary Information Secs. 5 and 6).

To reveal the sign-change feature in the edge states, we use a two-points pumping scheme. The feed source from a vector network analyzer Agilent E8363A is divided into two branches by power divider and connected to the two feed probes (indicated by the red asterisks in Fig. 3b) through a pair of Gore phase stable cables, which guarantees that the two feed probes have nearly the same signal (including magnitude and phase). The sign-change picture in Fig. 1b is confirmed by the finite-element simulation of the edge states for the dislocation structure with an inserted chunk of topologically trivial PhC (see Fig. 3c): on the left (right) side of the dislocation the two edge waves are of opposite (the same) phases. Such a sign-change feature, being a smoking-gun signature of the Dirac mass domain-wall, is confirmed in our experiments using the two-points pumping scheme (Fig. 3d). Although each feed probe pumps only one of the edge states, the relative phase of the two edge states clearly indicates the *π* phase elapse induced by the dislocation.

**Fig. 4:**
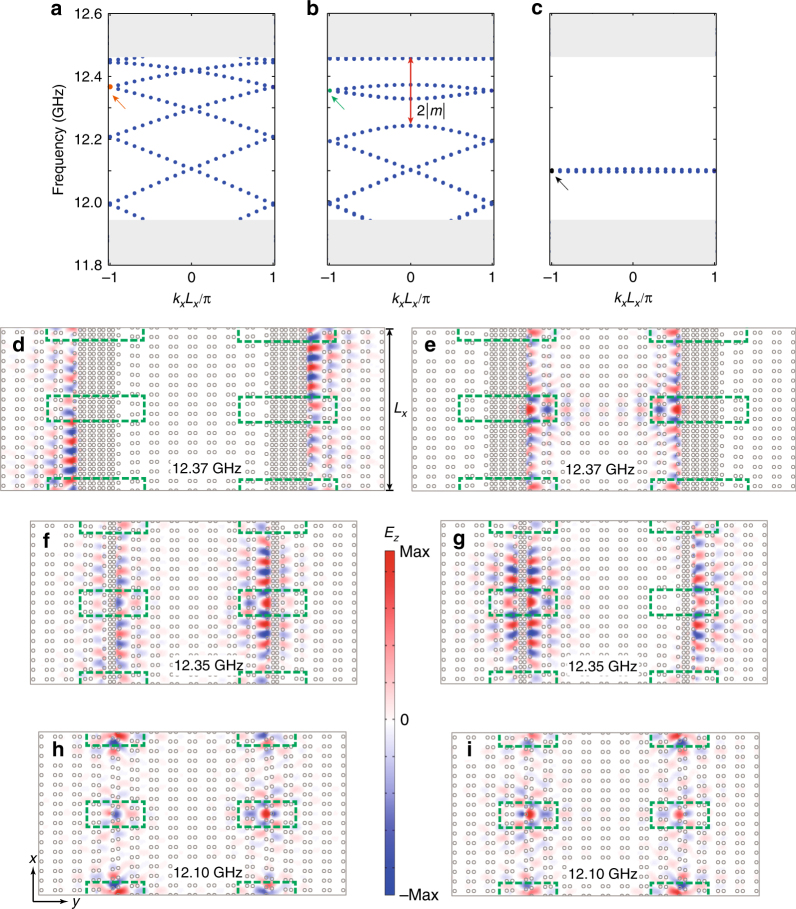
Electromagnetic simulation of the gluing process. **a**–**c** Photonic spectrum during the gluing process with decreasing width of the chunk of the topologically trivial photonic crystal where $L_x \!\! \prime = 4a_y \!\! \prime ,a_y \!\! \prime ,0$, respectively. The shaded regions represent the bulk photonic bands. Within the bulk photonic band gap, there are photonic states associated with edge states and localized states bound to the dislocations. **d**, **e** The field profiles of the degenerate photonic states labeled by the red arrow in **a**. **f**, **g** (**h**, **i**) The field profiles for the degenerate states labeled by the green (black) arrow in **b** (**c**). The green boxes enclose the dislocations

To further verify the cut-and-glue picture, we study the spectrum and field profiles of the photonic states within the PBG II during the gluing processes using finite-element simulations. To employ periodic boundary conditions in the finite-element simulation, we use a supercell with two chunks of trivial PhCs and four dislocations (indicated by the green boxes in Fig. 4) to calculate the eigen-spectrum and the field profiles. The Burgers vectors of the four dislocations are summed up to zero which is crucial for the periodic boundary condition. The supercell has a length of *L*_*x*_ along the *x* direction, while the length along the *y* direction depends on the thickness of the two chunks of topologically trivial PhCs *L*_trivial_. We start from the situation with $L_{{\mathrm{trivial}}} = 4a_y \!\! \prime$ where the photonic spectrum in Fig. 4a indicates merely band-folding of the edge states on all the boundaries. The field profiles in Fig. 4d and e show that the two opposite edge channels are nearly independent and uncoupled. When the thickness is reduced to $L_{{\mathrm{trivial}}} = a_y \!\! \prime$, the coupling between opposite edge channels become considerable. As shown in Fig. 4b, the spectrum of edge states no longer spans the whole PBG, a small gap is developed due to such coupling. The magnitude of this gap is equal to 2|*m*| where *m* is the Dirac mass for the edge states. Within this edge gap there are photonic states which are weakly localized on the dislocations, i.e., the Jackiw–Rebbi modes. The localization lengths are comparable with *L*_*x*_/2, as indicated in the electric field distributions in Fig. 4f and g. Hence, the spectrum of the Jackiw–Rebbi states is still dispersive, due to considerable coupling between nearby Jackiw–Rebbi modes. The field profiles in Fig. 4f and g indicate strong mixing between opposite edge channels as well as weak field localization on the dislocations. Finally, for *L*_trivial_ = 0, the in-gap photonic spectrum becomes nearly flat (Fig. 4c), indicating strongly localized states. Indeed, the electromagnetic fields are entirely localized on the dislocations, as shown in Fig. 4h and i. In this case, all the edge states are moved into the bulk bands, since the two chunks are entirely removed from the structure. In this way, the cut-and-glue picture is completely manifested in electromagnetism through finite-element simulation of the Maxwell equations.

### Experimental observation of the topological cavity mode

**Fig. 5:**
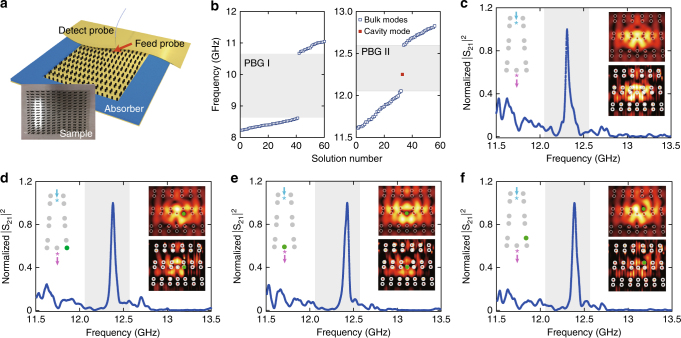
Robust topological cavity mode on a dislocation. **a** Experimental setup: the photonic crystal with a dislocation is cladded above and below by aluminum plates and mounted onto a 2D mapping system. **b** Bulk and cavity modes from simulation for PBG I (left) and PBG II (right). There is only one cavity mode in PBG II, whereas no cavity mode in PBG I. **c**–**f** Transmission and field profiles (upper insets: simulation, lower insets: experiments). The left insets show the structure near the dislocation: the gray points represent the YIG pillars and the green points denote metallic pillars. The upper arrows indicate electromagnetic wave injection at the cyan asterisk point, while the lower arrows indicate electromagnetic wave detection at the purple asterisk point in transmission measurements

The experimental setup for the dislocation structure and the measurement scheme is shown in Fig. 5a. The electromagnetic wave is excited through the feed probe near the bottom cladding and detected by the detect-probe near the top cladding. The feed probe is fixed at a position near the dislocation, while the detection position is changeable. Both the amplitude and the phase of the local electromagnetic fields are scanned using a 2D mapping system in a frequency-resolved manner (see Methods).

Our finite-element simulation study indicates that there is no localized state in the topologically trivial PBG between the second and the third bands (denoted as PBG I, 8.12–10.17 GHz), whereas there is one cavity mode in PBG II (topologically nontrivial PBG, 12.05–12.60 GHz) which is localized on the dislocation (see Fig. 5b). This observation confirms that the emergence of the mid-gap cavity mode is solely due to the dual-topology mechanism: without the nontrivial topology in wavevector space, the dislocation alone cannot induce 0D light-trapping. The electromagnetic field profile of the cavity mode indicates strong light-trapping on the dislocation with a localization length *l*_loc_ = 1.0*λ* (estimated from $l_{{\mathrm {loc}}} = \sqrt {A/\pi }$ where *A* is the Gaussian modal-area). Experimentally, we measure the transmission between two points which are located at different sides of the dislocation (inset of Fig. 5c). The transmission has only one peak at 12.37 GHz in the PBG II (the shaded regions in Fig. 5c–f), indicating only one localized mid-gap mode. The mode profile measured at the peak frequency in experiments is comparable with the field profile of the topological cavity mode from the finite-element simulation (see the insets of Fig. 5c).

We now study the transmission and the field profiles when the dislocation structure is perturbed. For instance, we replace one of the YIG pillar close to the dislocation by a metallic pillar of the same size. As shown for the cases in Fig. 5d–f, the topological cavity mode is found to be resilient against such perturbations. For all these cases, there is only one peak in the transmission spectrum in the PBG II, which reflects the robustness of the cavity mode and the topological light-trapping mechanism (for simulations comparable with the experimental results, see Supplementary Note 7). From the experimental observations, we notice that the frequency of the topological cavity mode is also stable against perturbations. The peak frequencies in Fig. 5d–f are 12.43, 12.50, and 12.43 GHz, respectively (corresponding to a relative change of 0.5%, 1%, and 0.5% compared to the unperturbed one, separately). A comparative study with conventional photonic cavities formed by defect modes in a PhC with a comparable PBG (but topologically trivial) shows that the frequency of the topological cavity mode is more stable than the conventional PhC cavities (see Supplementary Note 8). For the latter system, the number of cavity modes can also be modified when a nearby dielectric pillar is replaced by a metallic pillar of the same size, whereas there is always one topological cavity mode due to the dual-topology mechanism in the PBG. This observation is another evidence that the topological cavity mode is more stable than conventional PhC cavities.

## Discussion

Achieving topological light-trapping at *d*-2 dimensions in a *d*-dimensional photonic system opens up the possibility of realizing many new physical effects and applications. One possible application in photonic circuitry, a waveguide-coupler which couples two chiral edge channels through the topological cavity mode, is demonstrated in the Supplementary Note 9. The lower-dimensional topological light-trapping unveiled in this work gives rise to several important open questions: whether dual-topology can be exploited to induce wave localization in non-Hermitian or nonlinear systems where topological lasing^28,47–49^, parity-time symmetry, and other important effects can emerge^50^; what are the consequences when such topological cavity modes are coupled with quantum dots or other single-photon emitters; how to extend to higher dimensions, such as a topological waveguide induced by a 1D dislocation line in a 3D PhC; can the dual-topology mechanism be extended to quasi-periodic photonic systems^23–26^ where higher-dimensional physics can be simulated in low-dimensions? All these questions can lead to interesting physics and applications in the future. Our work paves the way toward lower-dimensional topological wave localization through dual-topology.

## Methods

### Materials and sample fabrication

All the samples in this paper are fabricated by the low loss commercial YIG ferrite pillars. The saturation magnetization is measured as 4*πM*_s_ = 1884 G by a vibrating sample magnetometer and relative permittivity is retrieved as 15.26–0.003*i* by the transmission/reflection method which can be treated as a constant at the microwave frequencies of interest (i.e., the PBG II). The fired ferrite is machined into pillars with a radius of *R* = 2 mm and height *h* = 10 mm. The topologically trivial PhC is realized by reducing the lattice constants to $a_x \!\! \prime = a_x/2$ and $a_y \!\!\prime = a_y/2$, as well as *d* → *d*′ = 5 mm, while keeping the radius, height, and material of the pillars unchanged.

### Experimental setup and measurement

The measurement setup for the topological dislocation, as schematically shown in Fig. 5a, consists of a vector network analyzer Agilent E8363A, a 2D mapping system, in a structure with top and bottom metallic cladding using aluminum plates. The upper (fixed) metallic plate has an area of 1 × 1 m^2^. The lower metallic plate (movable) has an area of 0.5 × 0.5 m^2^. The mapping area can be as large as 0.5 × 0.5 m^2^ when a single detect-probe is used. An array consisting of 364 permanent magnet NdFeB pillars are embedded into a 3-mm-thick aluminum plate (the lower metallic plate) in the sample with the dislocation. This plate works simultaneously as the metallic cladding as well as the external magnetic field source. Each NdFeB pillar is of radius 1.5 mm and height 3 mm. It can induce maximally 2800 Oe surface magnetic field. These NdFeB pillars apply one-to-one external magnetic field to the YIG pillars set between the magnet plate and top aluminum plate. Since the NdFeB pillars are outside of the metallic cladding, they do not affect the electromagnetic waves inside the cladding, except providing the magnetic fields. On average, the NdFeB pillars provide an external magnetic field of about 900 Oe. The whole photonic structure is surrounded by microwave absorbers. The field profiles are measured by scanning the electromagnetic fields through changing the position of the detect-probe.

### Band structure and simulation

The band structure and all the simulations were calculated by using the commercial software COMSOL MULTIPHYSICS with the RF module.

### Data availability

The data that support the findings of this study are available from the corresponding authors on reasonable request.

## Electronic supplementary material

Supplementary Information

Peer Review Report
