# Supplementary material for: Topological light-trapping on a dislocation
Source: Nat Commun. 2018 Jun 25;9:2462. doi: 10.1038/s41467-018-04861-x (PMC6018105; doi:10.1038/s41467-018-04861-x)
Supplement: Supplementary file 1 — Supplementary Information [file 41467_2018_4861_MOESM1_ESM.pdf]

Topological light-trapping on a dislocation

Fei-Fei Li *et al.*

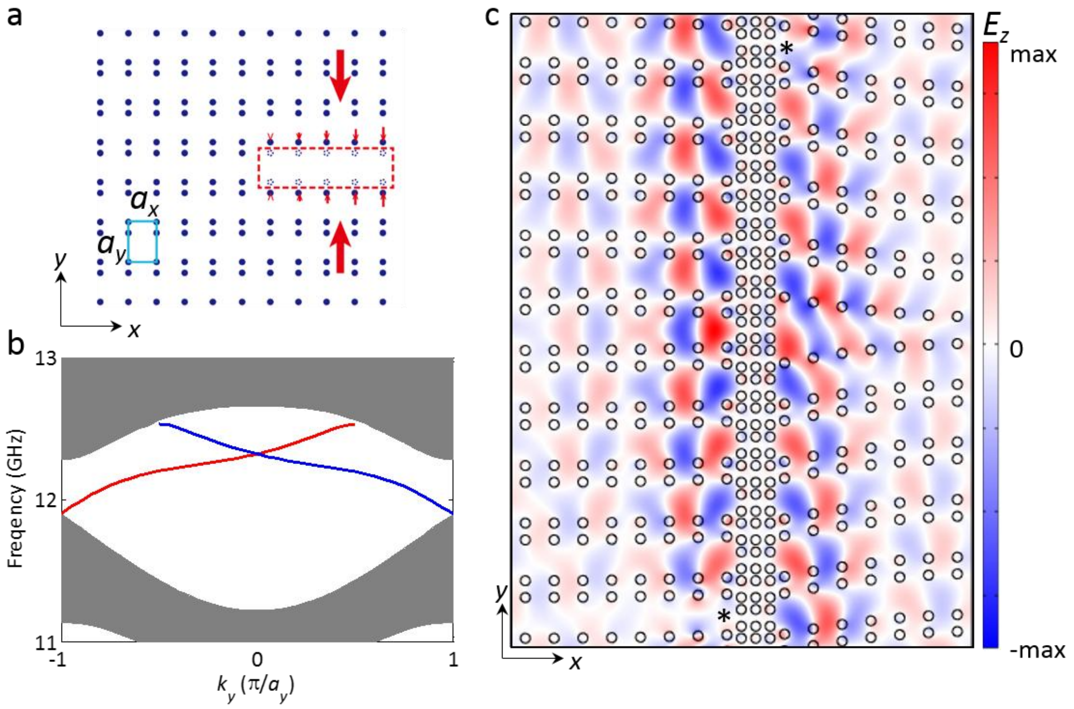

Supplementary Figure 1 | A dislocation with a Burgers vector $(\mathbf{0}, \boldsymbol{a_y})$. a, Schematic for the formation of a dislocation with a Burgers vector $(0, a_y)$, The blue rectangle shows the lattice constant $a_x(a_y)$ along the $x(y)$-direction. The dashed circles in the red rectangle refer to the removed lattice sites, while the arrows stand for the squeezing direction to form the dislocation. **b,** The edge (red and blue curves) and bulk (gray zones) spectrum of the topological photonic crystal with a boundary periodic along the y direction. The boundary is between the topological photonic crystal and the topologically trivial photonic crystal. **c,** The electric field pattern of the photonic edge states in the presence of the dislocation with the Burgers vector $(0, a_y)$. The electromagnetic fields are excited using two point-sources (labeled by the black asterisks). The relative phase between the two opposite edge channels does not change across the dislocation.

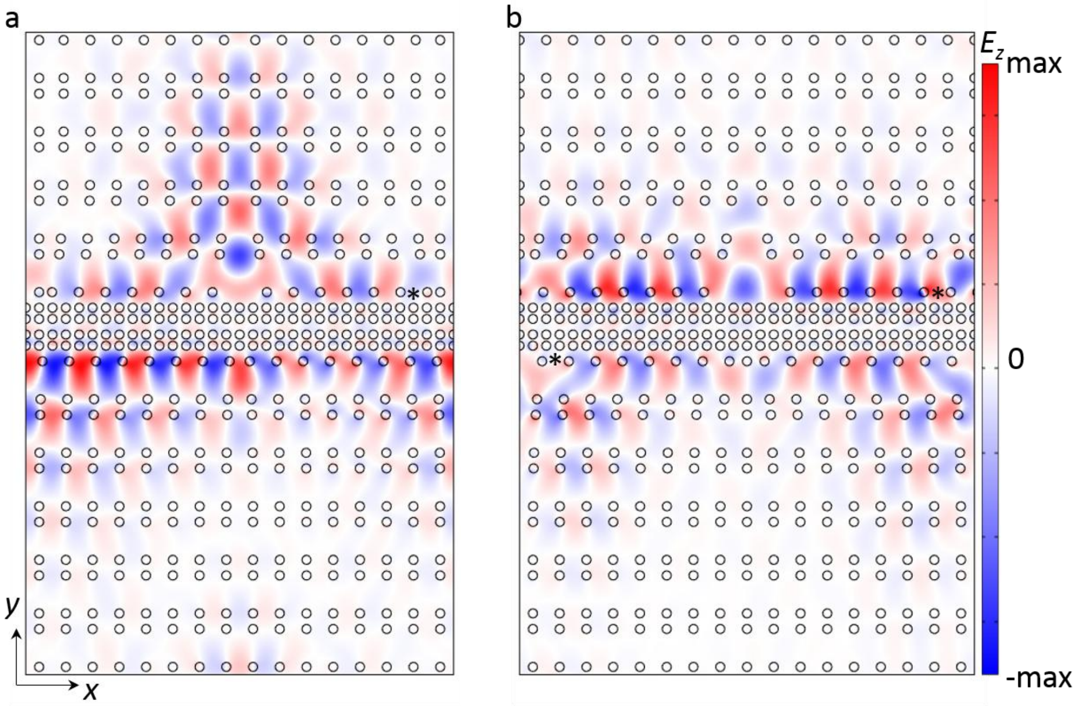

Supplementary Figure 2 | A dislocation with Burgers vectors $(2a_x, 0)$ and $(3a_x, 0)$. a, The electric field pattern of the photonic edge states in the presence of a dislocation with a Burgers vector $(2a_x, 0)$. The relative phase between the two opposite edge channels does not change across the dislocation. **b,** Field pattern of the photonic edge states in the presence of a dislocation with a Burgers vector $(3a_x, 0)$. The relative phase between the two opposite edge channels experience a π phase elapse across the dislocation. For both **a** and **b**, the electromagnetic fields are excited using two point-sources (labeled by the black asterisks).

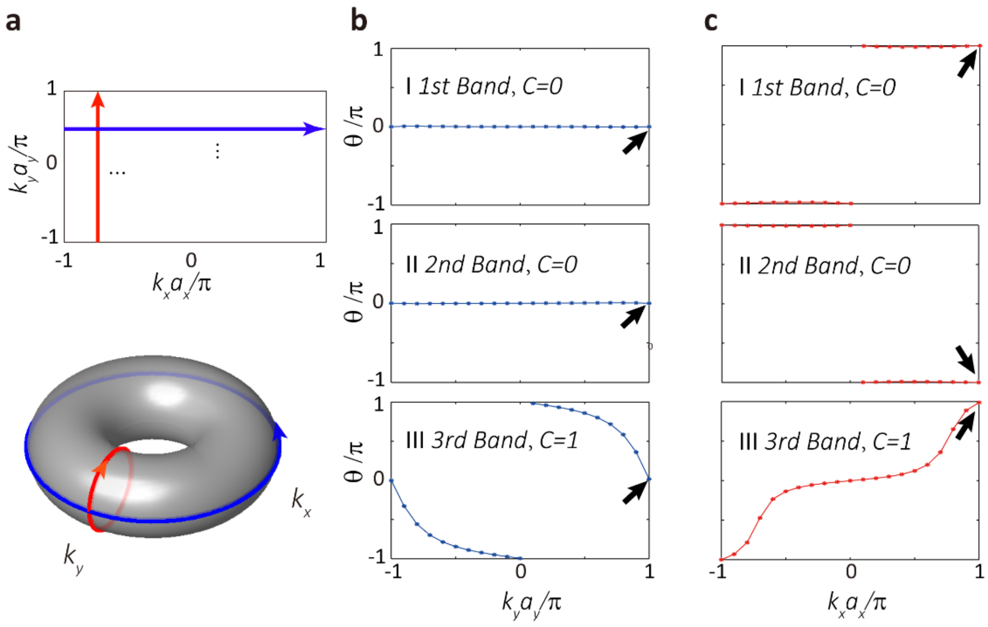

Supplementary Figure 3 | Calculation of the Zak phase and Chern number through the Wilson-loop approach. a, Equivalence of the 2D Brillouin zone and a torus, red (blue) line represents the closed-loop for the calculation of the Zak phase with a fixed k_x (k_y). **b** and **c**, Evolution of Zak phases for the (I)1st , (II)2nd and (II)3rd band on the torus with **(b)** k_y and **(c)** k_x, respectively. The Zak phases of the lowest three bands at $k_y = \pi/a_y$ are all equal to 0, as indicated by the black arrows, whereas the Zak phases of the lowest three bands at $k_x = \pi/a_x$ are all equal to π, as indicated by the black arrows. The phase accumulation of the Zak phases θ gives the Chern number for the first three bands as $C = 0, 0, 1$, separately.

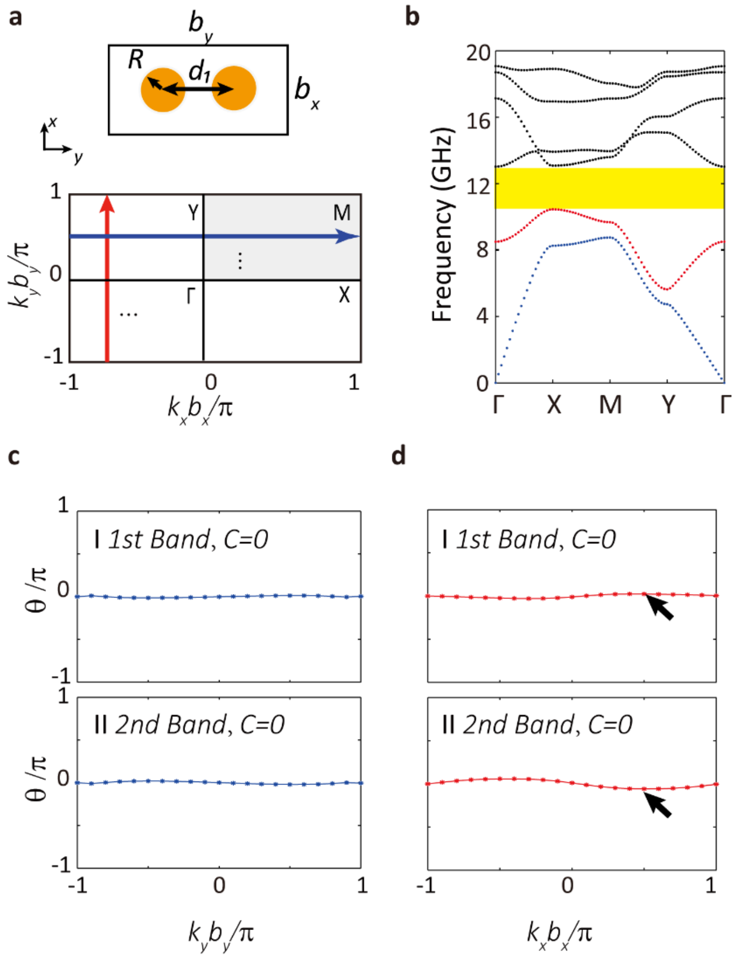

Supplementary Figure 4 | Calculation of the Zak phase and Chern number through the Wilson-loop approach for topological trivial photonic crystal. a, Unit cell of the topologically trivial photonic crystal and the Brillouin zone. **b,** Photonic band structure of the topologically trivial photonic crystal. The yellow area refers to the topologically trivial photonic band gap, which covers the topological photonic band gap in the main text. **c,** Evolution of the Zak phases of the (I)1st and (II)2nd photonic bands on the torus with fixed k_y. **d,** Evolution of the Zak phases of the (I)1st and (II)2nd photonic bands on the torus with fixed k_x. The black arrows indicate the Zak phases at $k_x = \pi/(2b_x) = \pi/a_x$ for the first two photonic bands.

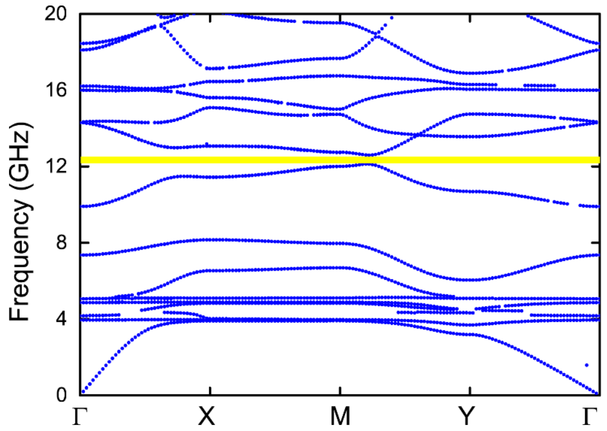

Supplementary Figure 5 | Photonic band structure calculated using dispersive permeability via multiple scattering numerical methods. The yellow shaded region refers to the topologically nontrivial photonic band gap.

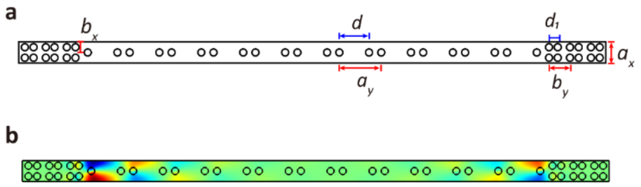

Supplementary Figure 6 | Supercell geometry construction for edge states calculation. a, Construction of the photonic supercell including 11 unit-cells of the topological photonic crystal and 12 unit-cells of the topologically trivial photonic crystal. The labels of $a_x, a_y, d(b_x, b_y, d_1)$ are geometry parameters for the topological nontrivial (trivial) photonic crystal, which are the same as mentioned above. **b,** Eigen electric field distribution of the supercell at $k_x = \pi/a_x$ which corresponds to the frequency 12.55 GHz.

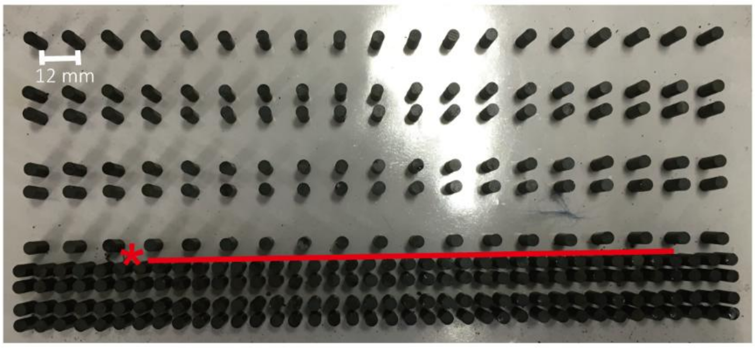

Supplementary Figure 7 | Sample for the measurements of the chiral edge states and their non-reciprocal photon propagations. The red asterisk refer to the feed probe and the sample is placed in a parallel plate waveguide with a detect probe sliding along the red line.

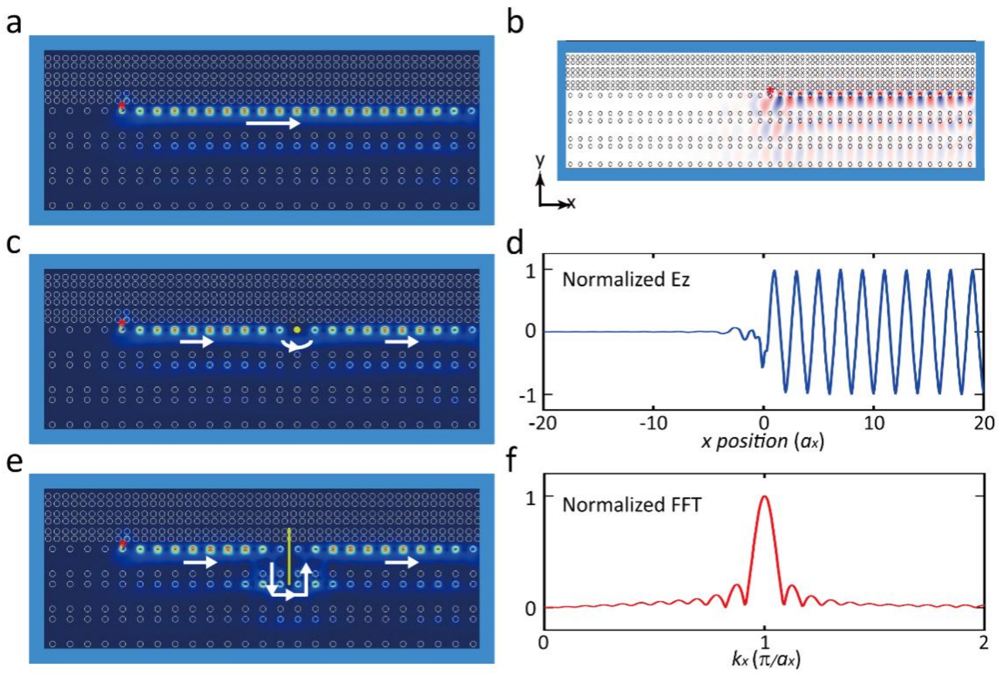

Supplementary Figure 8 | The unidirectional propagation properties of the chiral edge states. **a**, **c**, **e**, Simulated power flows when a point source (red star) oscillates at 12.55 GHz. **a** represents the perfect boundary between the topological photonic crystal and the trivial photonic crystal, **c** corresponds to the case where a YIG pillar (the yellow dot) is replaced by a metallic pillar of the same size, **e** represents the case where a metal slit (the yellow line) is inserted to perturb the energy flow along the edge channel. The white arrows show the power flow direction. **b**, 2D field-profile of the edge states excited at the frequency 12.55 GHz. **d**, Distributions of the electric field E_z as a function of x along the edge for the frequency 12.55 GHz. **f**, The corresponding normalized Fourier transformation of the E_z profile in **d**.

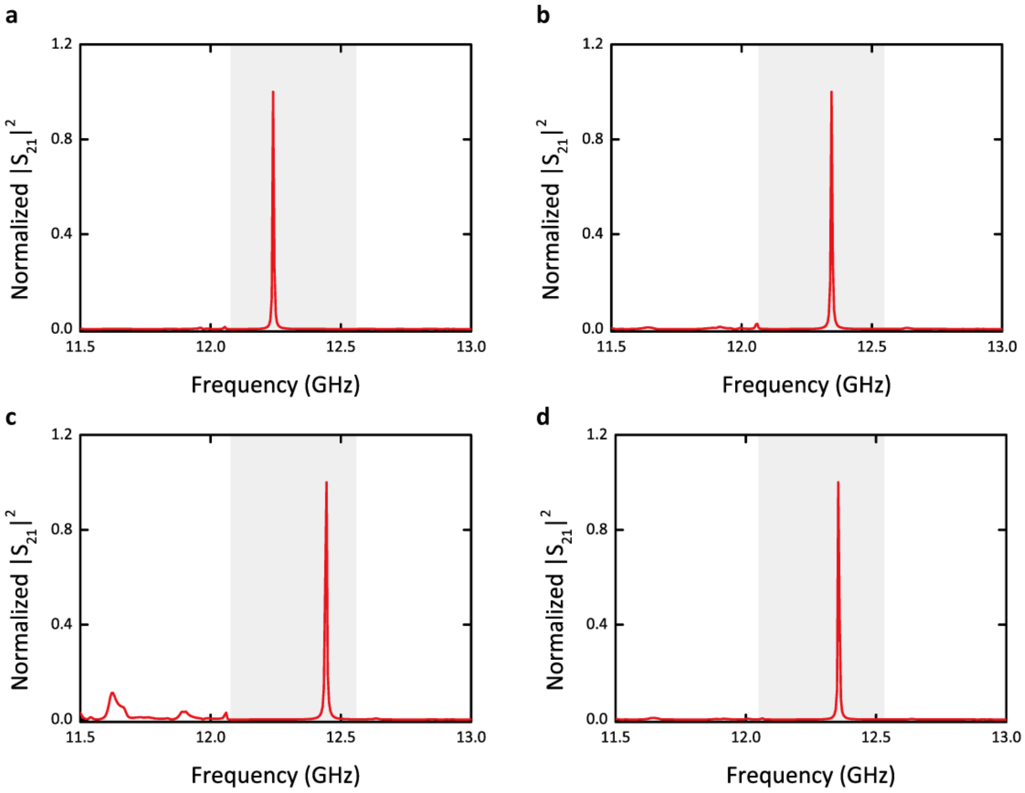

Supplementary Figure 9 | Finite-element simulation of the transmission across the dislocation as comparisons with experiments. The set-ups for the transmission are identical as those in Figure 5 in the main text. **a**, The transmission spectrum for the unperturbed dislocation structure corresponding to Fig. 5c in the main text. **b, c** and **d** are the transmission spectra for the perturbed dislocation structures corresponding to Figs. 5d, 5e and 5f in the main text, separately. For all these cases there is only one peak in the transmission in the topological band gap (the shaded regions), in agreement with the experiments. The peak frequencies in **a**, **b, c** and **d** are 12.29 GHz, 12.40 GHz, 12.50 GHz and 12.42 GHz.

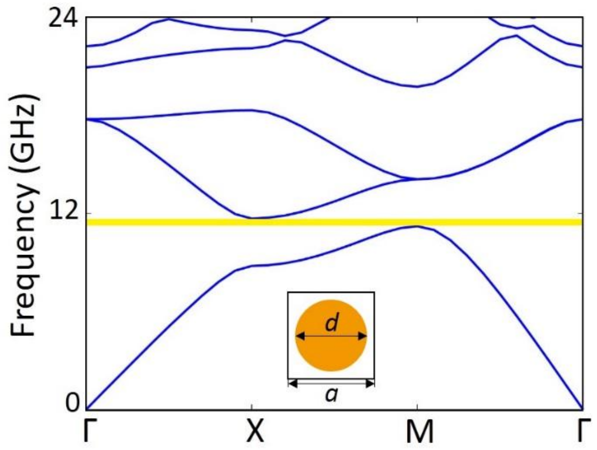

Supplementary Figure 10 | Band structure of the normal photonic crystal. Inset: the structure of the unit-cell. The photonic crystal is a 2D square lattice of dielectric rods. The lattice constant is $a = 0.5\,\text{cm}$. The diameter of dielectric rod denoted by orange circle is $0.824a$. The relative permittivity is 15.26, while the relative permeability is 1. The bandgap between 1st and 2nd band ranges from 11.21 to 11.68 GHz indicated by the orange region (the gap-to-mid-gap ratio is 4.1%, the same as for our topological photonic crystal).

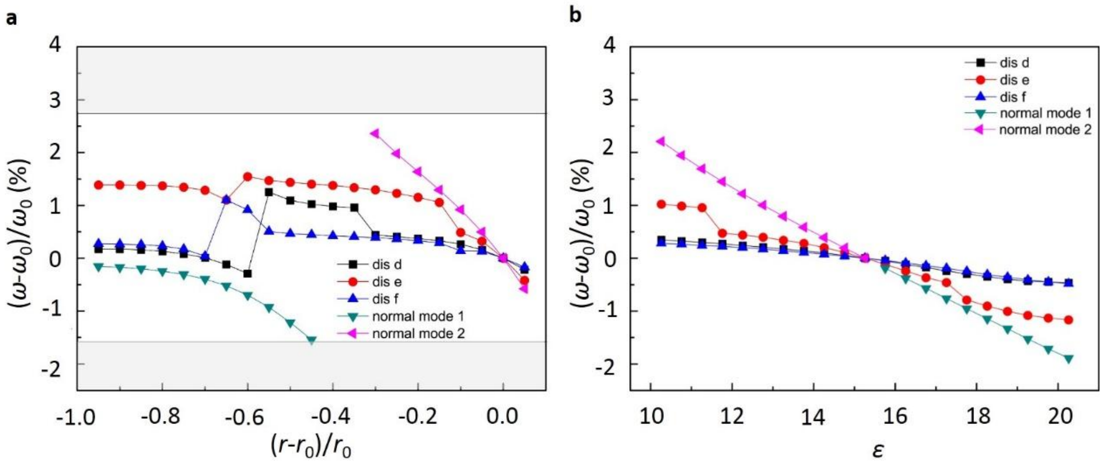

Supplementary Figure 11 | Robustness comparison between the topologically localized state on the dislocation and defect mode on the normal photonic crystal. Relative frequency change for the topologically localized mode on the dislocation and that for the defect mode in the normal photonic band gap material for **a** when the radius of a nearby dielectric rod is changed and **b** when the permittivity of the nearby dielectric rod is varied. The gray regions in **a** stand for the bulk bands for the conventional photonic crystal which hosts the normal defect modes.

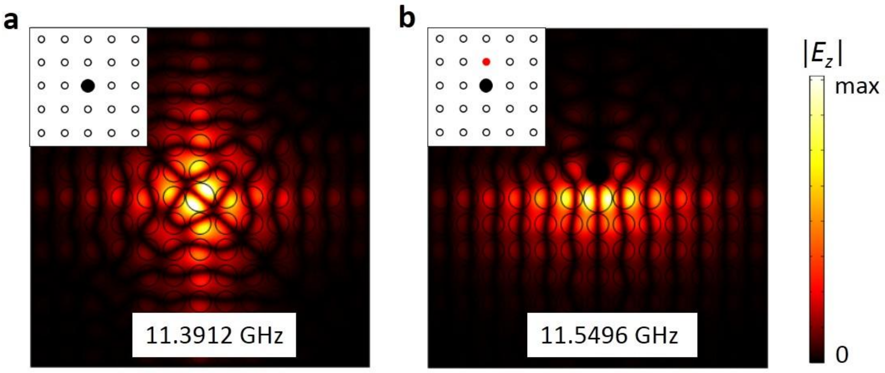

Supplementary Figure 12 | The defect states in the normal photonic crystal. Field patterns and frequency of the defect state for **a** an unperturbed defect mode **b** for a perturbed defect mode. The latter is realized by replacing a nearby dielectric rod (the red dot in the inset) is replaced by a metallic rod. The calculation is for the defect mode in the normal photonic band gap material.

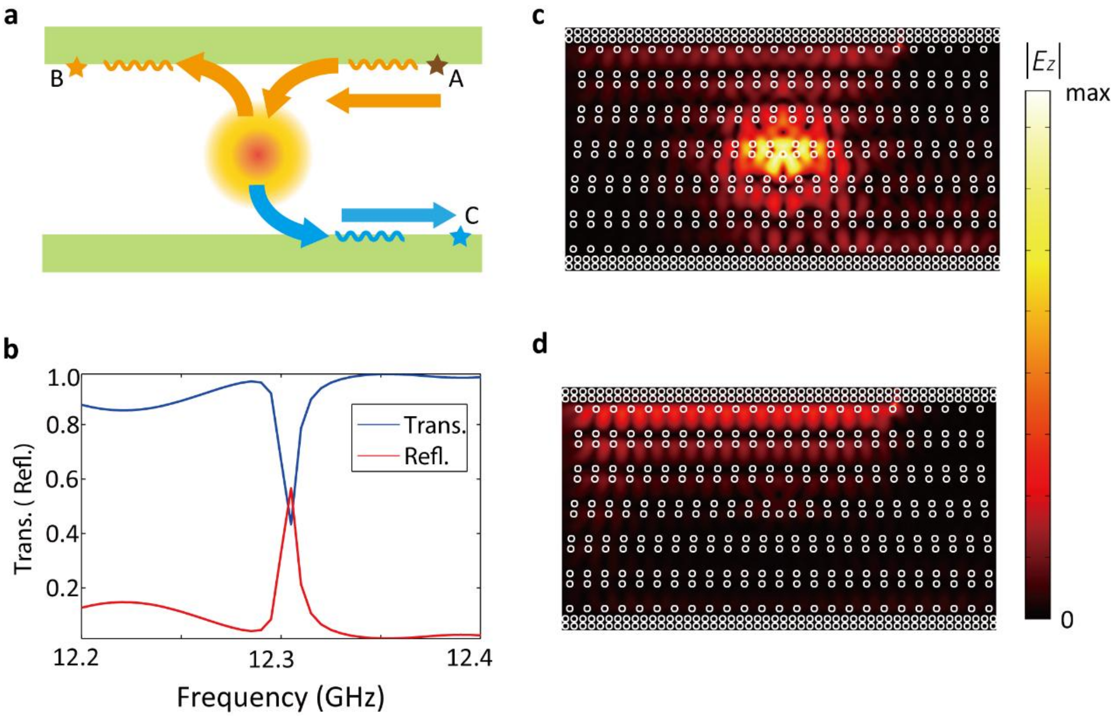

Supplementary Figure 13 | Waveguide coupler based on topological cavity mode. a, Schematic of the waveguide coupler based on the topological cavity mode. **b,** Transmission and reflection spectrum for the waveguide coupler where transmission means from A to B and reflection represent from A to C. **c** and **d,** The field-profiles from the finite-element simulation near the cavity resonance. **c,** At resonance, the upper edge channel couples efficient with the lower edge channel through the cavity mode. **d,** Off the resonance, most of the electromagnetic energy excited at point A are transmitted along the upper edge.

Supplementary Note 1 | Symmetry, Zak phase and edge Hamiltonian

There are four basic geometry and time-reversal operations in the system: inversion P, mirror operations M_x and M_y, and time-reversal operation T. The definition of time-reversal operation here also includes the reversal of the magnetic field and magnetization. Likewise, the mirror operations also include the reversal of the magnetic field and magnetization. Such a time-reversal operation, leaves the Maxwell equations (as well as the photonic spectrum) unchanged, because the permeability tensor μ is invariant under such a transformation. Similarly, the mirror operations also leave the Maxwell equations unmodified. Therefore, these four operations are symmetry operations.

Because of the inversion symmetry, the dispersions of the edge states at two opposite boundaries (for boundaries along the x direction) are related by the transformation: $k_x \to -k_x$, $\omega \to \omega$. Therefore, the intersection point of the dispersions of the two opposite edges can only located at $k_x = 0$ or $k_x = \pi/a_x$. Since the Zak phase π at $k_x = \pi/a_x$ leads to deterministic emergence of edge states there [3], such a crossing point can only be the $k_x = \pi/a_x$ point, if there is only one chiral edge state on a single boundary. Since in our photonic crystal the Chern number is 1 and the Zak phase at $k_x = \pi/a_x$ is π, this is the exactly the situation. The intersection point of the two edge dispersions is hence at the $k_x = \pi/a_x$ point, which is confirmed by numerical finite-element simulation, as shown in the main text.

The only form of the Hamiltonian of the coupled edge states that fulfills the four symmetries is $H = vq_x\sigma_z + m\sigma_x$ (v and m are real numbers). Since $\sigma_z = \pm 1$ denotes the two opposite edges, the inversion operation is then manifested as $P \coloneqq \sigma_x \otimes (q_x \to -q_x)$. The two mirror operations for the edge states are manifested as $M_y \coloneqq \sigma_x \otimes (v \to -v)$ and $M_x \coloneqq (q_x \to -q_x) \otimes (v \to -v)$. The time-reversal operation is $T \coloneqq (q_x \to -q_x) \otimes (v \to -v) \otimes K$ where K is the complex conjugation. From these symmetry operations, one finds that mass terms proportional to σ_y are not allow.

With such a constrained Hamiltonian, the Jackiw-Rebbi theory applies to our photonic system. When there is a mass domain wall at $x = 0$ described by $m(x)$ with $m > 0$ for $x > 0$ and $m < 0$ for $x < 0$, the solution of the soliton mode is given analytically as $E_z(x) = A_N \exp[-\frac{1}{v}\int_0^x dx' \; m(x')] \binom{1}{-i}$ where A_N is the normalization coefficient for the electric fields E_z. The localization length of this soliton mode is determined by the magnitude of the Dirac mass, the group velocity and the size of the dislocation. The larger the Dirac mass is, the smaller is the localization length of the Jackiw-Rebbi soliton mode.

Supplementary Note 2 | Dislocations with other Burgers vectors

In this section, we discuss dislocations with other Burgers vectors and the existence/non-existence of localized photonic modes for such dislocations. According to Eq. (1) in the main text, there is only one topologically localized state on the dislocation, if and only if the x component of the Burgers vector is a_x (or an odd integer times of a_x).

We first check the situation when the Burgers vector is $(0, a_y)$. The dislocation with such a Burgers vector is formed by taking half a row of the unit-cells away and deform the photonic crystal to restore the lattice order everywhere except for the dislocation center (Supplementary Figure 1a). Due to the trivial Zak phase at the YM line, there is no guaranteed spectral crossing at $k_y = \pi/a_y$ between the edge states at opposite boundaries. In fact, we find that the spectra of the two opposite

edge channels cross each other at $k_y = 0$ (Supplementary Figure 1b). The relative phase between opposite edge channels does not change across the dislocation, since the phase accumulation due to extra lattice translation is zero. This feature is indeed confirmed by our numerical simulation in Supplementary Figure 1c, where the relative phase between opposite edge channels remains the same on the left and right sides of the dislocation. Therefore, such a dislocation cannot lead to the formation a Dirac mass domain-wall or the topologically localized Jackiw-Rebbi mode.

We now move on to study the dislocation with a Burgers vector of $(2a_x, 0)$. We first show, according to the field profile in Supplementary Figure 2a, that the phase difference for the opposite edges remain zero on both the left and right sides of the dislocation. This phenomenon results from the fact that the Burgers vector $(2a_x, 0)$ gives rise to 2π shift to the phase difference between the upper and lower edges, since $\pi/a_x \times 2a_x = 2\pi$. Therefore, there is no sign change in the Dirac mass of the edge states across the dislocation. Thus, there is neither Dirac mass domain-wall nor the associated Jackiw-Rebbi state.

For the dislocation with a Burgers vector of $(3a_x, 0)$, the relative phase between the two opposite edge channels experience a π phase elapse across the dislocation (see Supplementary Figure 2b), which agrees with the simple arithmetic $\pi/a_x \times 3a_x = \pi \pmod{2\pi}$. Such a π phase elapse leads to the formation of the Dirac mass domain wall according to electromagnetic perturbation theory. We thus confirmed the Z_2 nature of the Dirac mass domain wall and the associated Jackiw-Rebbi localized mode.

Finally, we remark that, a dislocation, as a structure defect, can also introduce normal (non-topological) defect modes. Indeed, in our calculation we find modes in the photonic band gap that are localized on the dislocation with a Burgers vector of $(2a_x, 0)$ which are due to non-topological mechanisms. Fortunately, for the dislocation studied in the main text, there is no such normal defect modes. The emergence of these normal defect modes is a feature of dislocations in photonic crystals. Since the photonic band gap is formed by multiple Bragg scattering, whenever a hole of considerable size is created in the photonic crystal, the emergence of normal defect states is not a surprise (similar phenomena are also admitted in Ref. [8]). These normal defect modes usually reside in the hole region, since the photonic crystal is acting as a mirror due to the photonic band gap. The smoking-gun feature of the topologically localized mode is the formation of the Dirac-mass domain wall. We have shown that such a feature is absent for the dislocation with a Burgers vector $(2a_x, 0)$. In contrast, the formation of the Dirac-mass domain wall is verified for the dislocation with a Burgers vector $(a_x, 0)$ in the main text. In comparison, in a tight-binding model, the number of states are determined by the number of lattice sites. There is no state emerging in the hole region where there is no lattice site. Therefore, there is no normal defect mode bound to the dislocation. Only the topologically localized mode can emerge in such a tight-binding model, as studied in Ref. [7].

Supplementary Note 3 | Calculation of the Zak phase and Chern number through the Wilson-loop approach

The Zak phase is calculated using the Wilson-loop approach. In photonic systems, the Zak phase for the nth band is defined as $\theta_n = \oint_\sigma \mathbf{\Lambda}_n \cdot d\mathbf{k}$, where σ refers to a closed loop in the Brillouin zone (see Supplementary Figure 3a) [4]. Specifically, σ refers to a closed loop with a given k_x or k_y (for instance, $k_x = \pi/a_x$ or $k_y = \pi/a_y$). Here, $\mathbf{\Lambda}_n = \frac{1}{2}[\mathbf{\Lambda}_n^E + \mathbf{\Lambda}_n^H]$ is the Berry connection

for the nth band, where $\mathbf{\Lambda}_n^E = -i\langle u_n^E(\mathbf{k})|\varepsilon(\mathbf{r})|\partial_\mathbf{k} u_n^E(\mathbf{k})\rangle$ and $\mathbf{\Lambda}_n^H = -i\langle u_n^H(\mathbf{k})|\mu(\mathbf{r})|\partial_\mathbf{k} u_n^H(\mathbf{k})\rangle$ are the contributions from the electric and the magnetic fields, respectively [5]. Here $u_n^E(\mathbf{k})$ and $u_n^H(\mathbf{k})$ are the periodic part of the Bloch wave functions for the electric and magnetic fields, respectively ($\mathbf{k}$ is the wavevector). $\varepsilon(\mathbf{r})$ and $\mu(\mathbf{r})$ refer to the permittivity and permeability tensors, separately. To calculate the Zak phase numerically, we divide the closed-loop into N sections as separated by $k_1, k_2, \dots, k_N$ ($k_{N+1} = k_1$). The calculation converges for a large enough N (for instance, $N = 20$). The partial derivatives and integrations are then substituted by finite differences and summations, respectively. In this way the Zak phase can be rewritten as: $e^{-i\theta_n} = \prod_{\mathrm{i}=1}^{N} \frac{1}{2}[\langle u_n^E(\mathbf{k}_\mathrm{i})|\varepsilon(\mathbf{r})|u_n^E(\mathbf{k}_{\mathrm{i}+1})\rangle + \langle u_n^H(\mathbf{k}_\mathrm{i})|\mu(\mathbf{r})|u_n^H(\mathbf{k}_{\mathrm{i}+1})\rangle]$. In the calculation, we obtain the electromagnetic field distributions for each $\mathbf{k}$ point using the COMSOL Multiphysics, a commercial package based on finite element method. By calculating the overlap integral of the electromagnetic fields, we obtain the Zak phase θ_n by taking the imaginary part of the logarithmic of the serial multiplication.

The Chern number can also be calculated using the Wilson-loop approach [6]. The nonzero Chern number C originates from the nontrivial Berry curvature. They are related by the following equation,

$$2\pi C = \int_{BZ} \nabla_\mathbf{k} \times \mathbf{\Lambda}_n \cdot d\mathbf{S} \tag{1}$$

Here $d\mathbf{S}$ is the area element vector in the wavevector space (i.e., the Brillouin zone which is a torus). According to Ref. [6], the above integration can be simplified as follows,

$$\begin{aligned} 2\pi C &= \int_{-\pi/a_x}^{\pi/a_x} dk_x \int_{-\pi/a_y}^{\pi/a_y} dk_y \left(\partial_{k_x}\Lambda_y - \partial_{k_y}\Lambda_x\right) \\ &= -\int_{-\pi/a_y}^{\pi/a_y} dk_y\, \partial_{k_y}\left(\int_{-\pi/a_x}^{\pi/a_x} dk_x\, \Lambda_x\right) \\ &= -\int_{-\pi/a_y}^{\pi/a_y} d\theta(k_y) \end{aligned} \tag{2}$$

Here $\theta(k_y) = \int_{-\pi/a_x}^{\pi/a_x} dk_x\, \Lambda_x$ is the Zak phase for a given k_y (i.e., the Berry phase for the closed loop in wave-vector space with a given k_y while k_x goes from $-\pi/a_x$ to π/a_x, see Supplementary Figure 3a). We calculate $\theta(k_y)$ for each k_y in the region $(-\pi/a_y, \pi/a_y)$, the Chern number is then manifested as *minus* the phase accumulation of $\theta(k_y)$ divided by 2π when k_y goes from $-\pi/a_y$ to π/a_y. The Chern number can also be obtained by calculating the Zak phases for each given k_x

$$\begin{aligned} 2\pi C &= \int_{-\pi/a_x}^{\pi/a_x} dk_x \int_{-\pi/a_y}^{\pi/a_y} dk_y \left(\partial_{k_x}\Lambda_y - \partial_{k_y}\Lambda_x\right) \\ &= \int_{-\pi/a_x}^{\pi/a_x} dk_x\, \partial_{k_x}\left(\int_{-\pi/a_y}^{\pi/a_y} dk_y\, \Lambda_y\right) \\ &= \int_{-\pi/a_x}^{\pi/a_x} d\theta(k_x) \end{aligned} \tag{3}$$

The difference is that, here the Chern number is equal to the phase accumulation of $\theta(k_x)$ divided by 2π when k_x goes from $-\pi/a_x$ to π/a_x.

As indicated by the arrows in Supplementary Figure 3b and 3c, the Zak phases of the lowest three bands at $k_y = \pi/a_y$ are all equal to 0, whereas at $k_x = \pi/a_x$ the Zak phases of the lowest three bands are all equal to π. The Chern numbers are obtained from the evolution of the Zak phases as a function of k_x or k_y. As shown in Supplementary Figure 3b and Supplementary Figure 3c, the Chern numbers for the first three photonic bands are 0, 0, 1, respectively. The corresponding winding phases as a function of k_y (k_x) are 0, 0, -2π (0, 0, 2π), separately.

We further calculate the Zak phases and Chern numbers for the topologically trivial photonic crystal. The topologically trivial photonic crystal is also a rectangular lattice which has the geometry parameters: $b_y = 2b_x = 12$ mm, $d_1 = 5$ mm and $R = 2$ mm (see Supplementary Figure 4a). These geometry parameters correspond to the reduction of the lattice constants by half, while keeping the radius of the YIG pillars unchanged. In this photonic crystal, the photonic band gap between the 2nd and 3rd band is from 10.45 GHz to 13.02 GHz (see Supplementary Figure 4b), which covers the topological photonic band gap (ranging from 12.05 GHz to 12.60 GHz) in the proposed photonic crystal in the main text.

To confirm that the Zak phases and Chern numbers are trivial for this photonic crystal, we calculate the Zak phases as functions of k_x and k_y. As shown in Supplementary Figure 4c and 4d, the Zak phases have no winding behavior in the Brillouin zone, hence the Chern number for the first and second photonic bands are both zero. We then find that the Zak phases for the first and second photonic bands are close to zero at $k_x = \pi/(2b_x) = \pi/a_x$ (see the black arrows in Supplementary Figure 4). More importantly, the summation the Zak phases the first two photonic bands is zero at $k_x = \pi/(2b_x) = \pi/a_x$. These observations conclude that both the Chern number and the Zak phase are trivial for the photonic band gap between the second and the third bands. Such an examination is necessary for our study on the cut-and-glue picture of the topological light-trapping on the dislocation.

Supplementary Note 4 | Photonic band structure with dispersive permeability

The Dirac point in Supplementary Figure 2a in the main text was gapped due to time reversal symmetry breaking, which can be achieved by applying an external dc magnetic field. It should be mentioned that the permeability we used in Supplementary Figure 2b in the main text is a constant tensor, where both μ_1 and κ are treated as constants, $\mu_1 = 0.95$, $\kappa = 0.43$ (i.e., dispersionless permeability). Such a treatment is a good approximation when studying the photonic bands in a small frequency range (e.g., for the photonic band gap with a nontrivial Chern number which is the focus of our study). Nevertheless, to confirm the validity of our study and conclusions, we recalculate the photonic band structure using the dispersive permeability [1],

$$\mu = \begin{pmatrix} \mu_1 & -i\kappa & 0 \\ i\kappa & \mu_1 & 0 \\ 0 & 0 & \mu_2 \end{pmatrix},$$

where

$$\mu_1 = 1 + \frac{\omega_m(\omega_0 + j\alpha\omega)}{(\omega_0 + j\alpha\omega)^2 - \omega^2}, \quad \mu_2 = 1, \kappa = \frac{\omega_m\omega}{(\omega_0 + j\alpha\omega)^2 - \omega^2}$$

Here $\omega_0 = \gamma H_0$ is the resonance frequency, H_0 is the effective bias magnetic field (i.e., a sum of the bias magnetic field and the demagnetization field), $\omega_m = 4\pi\gamma M_s$ is the characteristic frequency with M_s being the saturated magnetization, $\gamma = 2.8$ MHz/Oe is the gyromagnetic ratio and $\alpha = 0.003$ is the magnetic damping coefficient [1]. The permittivity constant is $\varepsilon = 15 - 0.003i$. The photonic band structure calculated using the above permeability via the multiple scattering method [2] is plotted in Supplementary Figure 5. The full photonic band gap is labeled by the yellow shaded area, which ranges from 12.11 GHz to 12.60 GHz (very close to the photonic band gap range 12.05 GHz - 12.60 GHz calculated by using the constant permeability, as shown in the main text). The nearly flat bands appeared in the low frequency regime are due to the spin resonance of the ferrite pillars and the Mie resonances.

Supplementary Note 5 | Calculation and measurement of edge states in a supercell geometry

We use the topologically trivial photonic crystal as the cladding material to construct the supercell together with the topological photonic crystal, as shown in Supplementary Figure 6a. In their common photonic band gap, there are only photonic edge states. The field distribution of these edge states (see Supplementary Figure 6b) are concentrated on the two boundaries between the topological trivial and nontrivial photonic crystals. From the finite-element calculation of the supercell, we obtain the dispersions of the photonic edge states and the projected bulk photonic bands, which are presented in Fig. 2c in the main text.

Supplementary Figure 7 show the sample fabricated for the measurement of the chiral edge states and their non-reciprocal propagations. A single boundary between the topological photonic crystal and the trivial photonic crystal is formed. The feed probe is marked as the red asterisk in the figure. The sample is placed in a parallel plate waveguide with a detect probe sliding along the red line. The whole structure (the sample and the cladding) is placed in a Helmholtz coil which generates a nearly uniform magnetic field of about 900 Oe. The electromagnetic fields of the edge modes are scanned with a spatial resolution of 4 mm. The forward and backward transmissions, S_{21} and S_{12}, are measured by switching the external magnetic field direction which is equivalent to reverse the direction of propagation.

Supplementary Note 6 | Electromagnetic wave propagation on the edge states

To verify the unidirectional propagation properties of the chiral edge states, we construct a strip sample with 3 layers of topological photonic crystal and 3 layers of trivial photonic crystal (see Supplementary Figure 8). The chiral edge states should emerge on the boundary between them.

We choose to work with the frequency of 12.55 GHz. The feed probe is marked as the red star in Supplementary Figure 8. Figures Supplementary Figure 8a, 8c and 8e show that the power flow of the electromagnetic wave is mainly confined at the boundary between the two photonic crystals. The electromagnetic wave propagates in a unidirectional manner: going from the left to the right, even there is an obstacle in the middle. The power flow can automatically circumvent the defect and keep unidirectional propagation, showing robustness against structural disorders.

In addition, we study the field profile as shown in Supplementary Figure 8b. If we define the coordinate of the feed probe in Supplementary Figure 8b as (0,0). Then we can plot the electric field E_z of the edge states at frequency 12.55 GHz as a function of x. It is clear in Supplementary Figure

8d that the electric field E_z is literarily zero in the region with $x < 0$, another visual evidence of unidirectional electromagnetic wave propagation along the edge states. Interestingly, after a Fourier transformation on the field profile of E_z, we find that there is only one main peak locating at $k_x = \pi/a_x$. This observation reveals that only one state is primarily excited once the frequency is fixed, another important consequence of unidirectional edge states.

Supplementary Note 7 | Finite-element simulation of the cavity mode on the dislocation

For the comparison with the experimental results in Fig. 5 in the main text, here we calculate the transmission spectrum across the dislocation using the same set-up via finite-element simulation using COMSOL Multiphysics. In this simulation, calculate the point to point transmission from the feed-probe to the detect-probe. The positions of these antennas are the same as in Fig. 5 in the main text. The original dislocation structure and those perturbed dislocation structures are considered. Here the results in Supplementary Figure 9a correspond to that for an unperturbed dislocation, whereas the transmissions in Supplementary Figure 9(b,c,d) correspond to that for perturbed dislocation where one of the YIG pillar close to the dislocation is replaced by a metallic pillar of the same size. The simulations of the four cases here correspond to the experimental measurements in Figs. 5c, 5d, 5e and 5f in the main text. It is seen that for all the four cases, there is only one peak in the transmission, indicating only one cavity mode on the dislocation. These results agree with the experimental discovery, demonstrating the robustness of the light-trapping mechanism due to the dual-topology against perturbations.

There are small deviations between the simulated peak frequencies and the measured peak frequencies, which is understood as mainly due to the inhomogeneous magnetic field in the experiments. The external magnetic field in the simulation is taken as uniform as 900 Oe, whereas in experiments it is realized by an array of self-biased ferrite made of NdFeB pillars which is incorporated in the aluminum cladding plates. Such a magnetic field is practically inhomogeneous. It can reach to an upper-bound value of 2800 Oe and a lower-bound value of 300 Oe. Overall it leads to an average effective magnetic field about 900 Oe. Such a difference makes the simulated peak frequencies deviates slightly from the measured peak frequencies.

Supplementary Note 8 | Robustness of the topologically localized state on the dislocation

In this section, we show that the number and frequency of the topologically localized state on the dislocation are much more robust than those of normal defect states in a photonic band gap. To do so, we compare our results with the calculations for the defect states in a normal photonic band gap material. The latter is a 2D square lattice photonic crystal consisting of dielectric rods of radius $r_0 = d/2 = 0.412a$ where the lattice constant is $a = 0.5$ cm (see Supplementary Figure 10). The permittivity of the dielectric rod is 15.26 (the same as that of the YIG rods). Such a photonic crystal has a photonic band gap between the first and the second bands with a gap-to-mid-gap ratio of 4.1%. This photonic band gap has trivial band topology. The mid-gap frequency and the gap ratio are comparable with our topological photonic band gap, which justifies the quantitative comparison between them.

The defect states in the normal photonic crystal is created by increasing the radius of one of the dielectric rod to $r_1 = 0.55a$ which generates a pair of (degenerate) defect states of p-wave

symmetry. The field profiles shown in Supplementary Figure 12, however, is a superposition of them, yielding a *d*-wave like shape. We test the robustness of the localized modes by three different types of perturbations: for type-I perturbations, we change the radius of a nearby dielectric rod; for type-II perturbations, we change the permittivity of this nearby dielectric rod; for type-III perturbations, we replace this nearby dielectric rod by a metallic rod. For our dislocation structure, there are three different choices of the nearby rod, which are indicated in Figs. 5d, 5e and 5f in the main text. For the defect structure in the normal photonic crystal, there are four nearby dielectric rods. However, due to C_4 rotation symmetry, perturbations on any one of them leads to the same results.

The main results of this section are summarized in Supplementary Figure 11 and Supplementary Figure 12. In Supplementary Figure 11a, it is shown that the relative change of the frequency of the topological cavity mode is considerably smaller than that of the normal defect modes when the radius of the nearby rod r_1 goes from $0.05r_0$ to $1.05r_0$. Particularly, when the relative change of the radius of the nearby rod is about -0.4, the normal defect mode 1 disappears (merges into the higher bulk band). For stronger changes of the radius of the nearby rod another normal defect mode 2 develops from the lower bulk band. In comparison, the relative change of the frequency of the topological cavity mode on the dislocation is smaller than 1.5% for the three different nearby rods, despite that the radius of the nearby rod has been significantly changed. This robustness is intrinsically because the appearance of the topological cavity mode is determined by the dual topology mechanism, which is irrelevant to the disorders.

The change of permittivity of the nearby rods also produce similar effects, as shown in Supplementary Figure 11b where the permittivity changes considerably from 10.26 to 20.26. The relative change of the frequency of the normal localized mode is considerably larger than that of the topologically localized mode. The same conclusion is arrived when we study type-III perturbations, i.e., replacing a nearby dielectric rod by a metallic rod. As shown in the main text, there is always one localized mode in the photonic band gap of which the frequency changes slightly. In contrast, for the normal defect states, such replacement leads to disappearance of one of the defect mode and considerable change of the frequency of the other mode (1.39%), as shown in Supplementary Figure 12. In comparison, the frequency shift of the topological cavity due to such replacement is smaller than 1%, meanwhile there is always one topological cavity mode in the PBG due to the dual topology mechanism.

Supplementary Note 9 | Waveguide coupler based on topological cavity mode

One of the potential applications of our topological cavity mode is a waveguide coupler in the integrated photonic chips. As shown in Supplementary Figure 13a, we sandwiched our dislocation structure with two topologically trivial photonic crystal slabs. Thus there are two boundaries with chiral edge states: in the upper edge electromagnetic waves can only propagate from right to left, whereas in the lower edge electromagnetic waves can only propagate from left to right. These two unidirectional waveguides cannot couple with each other unless through the cavity resonance in the middle.

Supplementary Figure 13a illustrates such coupling. Near the cavity resonance, the electromagnetic wave excited at the A point at the upper edge propagates from right to left. This wave may couple into the cavity mode and then transferred into the lower edge, or stay in the upper

edge. The former leads to wave propagation from the A point to the C point at the lower edge, the latter results in wave propagation from the A point to the B point at the upper edge. Thus, the functionality of the cavity mode is to split the beam excited at the A point into two beams, one beam still at the upper edge, the other beam is at the lower edge. Such a beam splitter is functioning near the resonance of the cavity mode. More than the beam splitter, the device is in fact a coupler between the two unidirectional waveguides, which allows signals to transfer between these waveguides.

In the simulation, the topological photonic crystal with a dislocation is chosen to have 6 layers between the two trivial photonic crystals. The transmission spectrum in Supplementary Figure 13b indicates clearly a resonance at 12.3 GHz, corresponding to the cavity resonance. The Fano line-shape is commonly seen in such a resonance. The transmission spectrum shows effective coupling between the two unidirectional waveguides near the cavity resonance. The field profile at the cavity resonance as shown in Supplementary Figure 13c indicates that the electromagnetic wave excited at point A can be efficiently coupled into the cavity mode and the lower edge channel. Away from the cavity resonance, as shown in Supplementary Figure 13d, the electromagnetic energy propagates mainly along the upper edge.

Supplementary References

[1] D. M. Pozar, *Microwave Engineering,* 2nd ed. Wiley, New York, 1998.

[2] S. Y. Liu, J. J. Du, Z. F. Lin, R. X. Wu, and S. T. Chui, Phys. Rev. B **78**, 155101 (2008).

[3] M. Xiao, Z. Q. Zhang, and C. T. Chan, Phys. Rev. X **4**, 021017 (2008).

[4] D. Xiao, M. -C. Chang, and Q. Niu, Rev. Mod. Phys. **82**, 1959 (2010).

[5] M. Onoda, S. Murakami, and N. Nagaosa, Phys. Rev. B **74**, 066610 (2006).

[6] H. M. Weng, R. Yu, X. Hu, X. Dai, and Z. Fang, Adv. Phys **64**, 227 (2006).

[7] Y. Ran, Y. Zhang, and A. Vishwanath, Nat. Phys. **5**, 298-303 (2009).

[8] L. Lu and Z. Wang, Topological one-way fiber of second Chern number. Preprint at http://arxiv.org/abs/1611.01998(2016).
