# Supplementary material for: Topological light-trapping on a dislocation
Source: Nat Commun. 2018 Jun 25;9:2462. doi: 10.1038/s41467-018-04861-x (PMC6018105; doi:10.1038/s41467-018-04861-x)
Supplement: Supplementary file 2 — Peer Review Report [file 41467_2018_4861_MOESM2_ESM.pdf]

Reviewers' comments:

Reviewer #1 (Remarks to the Author):

The paper reports the design and experimental observation of a localized mode on a dislocation in a two-dimensional photonic topological insulator at microwave frequencies. The underlying physics is dual-topology, i.e nontrivial topology in both the momentum space and the real space. It is shown that the number of the topological defect modes is determined by the second Chern number given by the dot product of the Berger's vector (real space) and a reciprocal vector described by the Zak phases along the two in-plane directions. The localized mode is also nicely explained by the change of the sign of the Dirac mass across the dislocation based on the Jackiw-Rebbi theory. Experimentally they observe the defect mode around the dislocation site, which is highly robust against presence of defects. This is a very timely and interesting work since only until very recently the phenomena arising from the second quantum Chern number were first experimentally investigated in photonics (see Nature volume 553, 59 (2018)). I am happy to recommend the publication of this work in Nature Communications once the following minor points are addressed.

1. How is the localization of dislocation mode affected by the separation between the two halves of the photonic crystal? The authors should investigate into the field patterns for different separations at least numerically.
2. It is interesting to see what would happen to the defect mode if the Zak phases of the XM and YM are swapped by rotating the crystal unit cell by 90 degrees while keeping the dislocation orientation fixed. Some simulation results on this configuration would be very helpful to clarify the importance of the Zak phases.
3. In order to confirm the Z2 nature of the mode, the authors should numerically study the field patterns for a number of different Burgers vectors (for example ranging from 1 to 3 times of the lattice vector along x direction).

Reviewer #2 (Remarks to the Author):

This work gives a comprehensive study of the topological defect modes associated with a dislocation in a 2D YIG photonic crystal. The work is complete with theory, simulation and experiment. While other papers on the similar topic of topological defect modes in photonic systems have appeared in the archive [e.g. arXiv:1611.02373; arXiv:1612.08687; arXiv:1611.01998] for sometime, this paper is the only work on dislocation-induced defect mode and it has experimental verification of the theory predictions. I think this paper has reached the level of novelty required in publication in Nature Communications. Before the paper can be accepted, I have some suggestions for the authors to consider.

First of all, the presentation can be improved. In the present form, the manuscript is written in a very concise manner. It allows the reader to get the main message: a defect mode exists in a specific defect (dislocation) of some specific 2D magnetic photonic crystals. However, if one wants to follow the argument and the details more closely, the reader has to go back and forth between the main text and the supplementary material. As there is no strict length restriction in Nature Communications, the authors may want to expand the text to make the reading the paper a less tedious task. This will be useful to readers in the wider field.

I also have some questions concerning Eq. (1), which is the key equation in this paper.

The vector Q in Eq. (1) is defined as $\vec{Q}=(\theta_{XM}/a_x, \theta_{YM}/a_y)$ where θ_{XM}, θ_{YM} are the Zak phases along XM and YM. The system is multi-band system, and it is not clear to me which band or bands these Zak phases should refer to. For a multi-band system, the topological character of the gap should be related to the Zak phases of all

bands below the gap (e.g. Ref [30] cited in the manuscript). Is the \vec{Q} here defined by the Berry connection of all the bands below the band gap, or just one band?

Equation (1) is used to count the number of localized modes, and it is a Z2 quantity according to the authors. According to the classification of topological defects (Ref.[38] cited in the paper), the topological defects characterized by a Z2 index considered in this manuscript should have Particle-Hole symmetry (see Table I of Ref. [38]). It is not obvious to me that the system has particle-hole symmetry. Can the authors elaborate on this point?

Reviewer #3 (Remarks to the Author):

In the present work the authors have used a concept called dual space topology to study a lattice system with dislocation. This mechanism allows one to trap light at a lower dimension. Stemmed from topological property, these localized modes are robust against perturbation, which the authors have studied theoretically and experimentally. The manuscript presents the first experimental realization of such topological dislocations and will be of interest to a wide range of researchers.
Overall, the paper is well organized in terms of theory and follows up with the corresponding experiment. All the theoretical claims were addressed and explained via reasonable experimental facts though it is lacking few details. But before recommending for publication, the following issues must be addressed:

1. It would be useful to clarify how this cavity mode is different from normal photonic crystal defect mode, e.g. in term of robustness to dielectric fluctuation. In other words, how does this system compare with a topologically trivial system, at least in a simulation? See comment 5.
2. Since the band gap is opening up between 3rd and 4th bands, the authors should specifiy the Chern number of 4th band so that the winding number of the edge state could be deduced.
3. Fig. 2c and d seem to be contradictory. The simulation in Fig. 2c shows the edge states spans from 12 to 12.5 GHz. But in experimental data of Fig. 2d the forward transmission is only present for the higher frequencies, around 12.5 GHz.
4. Is there any explanation for the forward transmission yellow region in the 13-13.5 HGz region. In principle there should not be any transmission because of the bulk modes, unless the system size is large compared to localized bulk modes. This seems to be not the case.
5. Why did the author only choose metallic defects and not just remove one of the YIG pillars? Is there any measure for the strength of the defect? How can one argue that something is a weak or strong perturbation, e.g. is it possible to compare the response to a topologically trivial system, as least in the simulation? In general, if one removes/perturbs a site in a photonic crystal the transmission is affected, but not completely vanished.
6. Have the authors measured/simulated the modification of the cavity mode frequency versus the defect strength? Intuitively, the transmission peak can move from fig-3-c to fig-3-d, while remaining in the bandgap.

Reply to the First Reviewer

Reviewer: “The paper reports the design and experimental observation of a localized mode on a dislocation in a two-dimensional photonic topological insulator at microwave frequencies. The underlying physics is dual-topology, i.e nontrivial topology in both the

momentum space and the real space. It is shown that the number of the topological defect modes is determined by the second Chern number given by the dot product of the Berger's vector (real space) and a reciprocal vector described by the Zak phases along the two in-plane directions. The localized mode is also nicely explained by the change of the sign of the Dirac mass across the dislocation based on the Jackiw-Rebbi theory. Experimentally they observe the defect mode around the dislocation site, which is highly robust against presence of defects. This is a very timely and interesting work since only until very recently the phenomena arising from the second quantum Chern number were first experimentally investigated in photonics (see Nature volume 553, 59 (2018)). I am happy to recommend the publication of this work in Nature Communications once the following minor points are addressed. (1). How is the localization of dislocation mode affected by the separation between the two halves of the photonic crystal? The authors should investigate into the field patterns for different separations at least numerically. (2). It is interesting to see what would happen to the defect mode if the Zak phases of the XM and YM are swapped by rotating the crystal unit cell by 90 degrees while keeping the dislocation orientation fixed. Some simulation results on this configuration would be very helpful to clarify the importance of the Zak phases. (3). In order to confirm the Z2 nature of the mode, the authors should numerically study the field patterns for a number of different Burgers vectors (for example ranging from 1 to 3 times of the lattice vector along x direction)."

Our reply: Please find one-to-one replies to the points raised by the reviewer below:

(1) We added investigations on the dependence of the localization of dislocation mode on the separation between the two halves of the photonic crystal, which is the current Fig. 4 in the main text. Associated with this Figure, we devoted a long paragraph in page 3 to discuss the underlying physics. Particularly, we show that as the width of the chunk of the topologically trivial photonic crystal is reduced, the edge states develop a mass gap, and eventually merge into the bulk bands when the chunk is removed. In the meanwhile, the Jackiw-Rebbi states become more and more localized, and eventually evolve into strongly localized states bound to the dislocation. This paragraph, together with the investigations on the sign-change feature of the edge states due to the dislocation, fully manifest the cut-and-glue picture in photonic crystals and the underlying physics of dual-topology in electromagnetism.

(2) Such a rotation is equivalent to study the dislocation with a Burgers vector of (0, a_y) since only the edge states along the *y* direction affect the underlying physics. We added the study of such a dislocation in the Supplemental Materials in Section 2. Because of the trivial Zak phase on the YM line, the spectra of opposite edge channels cross each other at k_y=0. Therefore, there is no phase accumulation between the opposite edge channels, and the relative phase difference between them does not change across the dislocation. As a consequence, there is no topological mode bound to the dislocation. The underlying physics is due to the trivial Zak phase on the YM line.

(3) We added simulation of field patterns for various Burgers vectors in the Supplemental Materials, according to the reviewer's suggestion. For Burgers vector of (2a_x, 0), the relative phase between the opposite edge channels experience a 2\pi=0 elapse across the dislocation, which does not lead to the Dirac mass domain wall. This is consistent with

our conclusion that a Burgers vector with even integer lattice translation along the x direction is equivalent to 0 Burgers vector which does not induce a Dirac mass domain wall or a topological cavity mode. For Burgers vector of ($3a_x$, 0), the relative phase between the opposite edge channels experience a 3\pi=\pi elapse across the dislocation. Therefore, there is a Dirac mass domain wall on the dislocation. These results confirm the Z2 nature of the Dirac mass domain wall and the associated Jackiw-Rebbi mode bound to the dislocation.

Reply to the Second Reviewer

Reviewer: "This work gives a comprehensive study of the topological defect modes associated with a dislocation in a 2D YIG photonic crystal. The work is complete with theory, simulation and experiment. While other papers on the similar topic of topological defect modes in photonic systems have appeared in the archive [e.g. arXiv:1611.02373; arXiv:1612.08687; arXiv:1611.01998] for some time, this paper is the only work on dislocation-induced defect mode and it has experimental verification of the theory predictions. I think this paper has reached the level of novelty required in publication in Nature Communications. Before the paper can be accepted, I have some suggestions for the authors to consider. First of all, the presentation can be improved. In the present form, the manuscript is written in a very concise manner. It allows the reader to get the main message: a defect mode exists in a specific defect (dislocation) of some specific 2D magnetic photonic crystals. However, if one wants to follow the argument and the details more closely, the reader has to go back and forth between the main text and the supplementary material. As there is no strict length restriction in Nature Communications, the authors may want to expand the text to make the reading the paper a less tedious task. This will be useful to readers in the wider field."

Our reply: We moved some of the important results in the supplemental material into the main text. For instance, the electromagnetic simulation of the cut-and-glue picture, which is now Fig. 4. We devote a long paragraph to discuss the evolution of the edge states and the dislocation localized states during the gluing process. This investigation, serving as a complementary to the sign-change feature, confirms the cut-and-glue picture in topological dislocation in photonics. We also strengthen the discussions on the frequency stability of the topological cavity mode. Besides, we added details on the formation and fabrication of the dislocation sample and the measurement details in the main text.

Reviewer: "I also have some questions concerning Eq. (1), which is the key equation in this paper. The vector Q in Eq. (1) is defined as \vec{Q}=(\theta_{XM}/a_x, \theta_{YM}/a_y) where \theta_{XM}, \theta_{YM} are the Zak phases along XM and YM. The system is multi-band system, and it is not clear to me which band or bands these Zak phases should

refer to. For a multi-band system, the topological character of the gap should be related to the Zak phases of all bands below the gap (e.g. Ref [30] cited in the manuscript). Is the \vec{Q} here defined by the Berry connection of all the bands below the band gap, or just one band? Equation (1) is used to count the number of localized modes, and it is a Z2 quantity according to the authors. According to the classification of topological defects (Ref.[38] cited in the paper), the topological defects characterized by a Z2 index considered in this manuscript should have Particle-Hole symmetry (see Table I of Ref. [38]). It is not obvious to me that the system has particle-hole symmetry. Can the authors elaborate on this point?”

Our reply: First, the Zak phases in Eq. (1) refer to the Zak phases of all bands below the gap. This is clarified in the revised manuscript. Second, the exact definition of the Z2 topology here indeed needs particle-hole symmetry, which is unrealistic for any physical systems, except for ideal superconductors within the mean field theory/approximation. In photonic crystals and many other classical wave systems, such symmetry does not exist. Breaking this symmetry weakens the topological protection of the cavity mode. However, if such symmetry breaking is not strong, the topological protection still exists and manifests in many aspects. Indeed, in our system we find that the topological cavity mode is robust against structural disorders, as demonstrated in our numerical simulation as well as experimental investigations. A comparative study shows that the frequency of the topological cavity mode is more stable than conventional photonic crystal cavity mode. In addition, the number of cavity mode due to the dual topological mechanism is robust, whereas the number of modes for conventional photonic crystal cavity can be modified if structural disorders and perturbations are introduced, as shown in our numerical simulation in Section 8 in the Supplemental Materials. Furthermore, the Z2 nature for the formation of the Dirac mass domain wall is manifested in the electromagnetic simulations in Section 2 in the Supplemental Materials: for a dislocation with a Burgers vector of an odd integer times (a_x, 0), there is a Dirac mass domain wall, whereas for a dislocation with a Burgers vector of an even integer times (a_x, 0), there is no Dirac mass domain wall; The Dirac mass domain wall also disappears for a dislocation with a Burgers vector of (0, a_y).

Reply to the Second Reviewer

Reviewer: “In the present work the authors have used a concept called dual space topology to study a lattice system with dislocation. This mechanism allows one to trap light at a lower dimension. Stemmed from topological property, these localized modes are robust against perturbation, which the authors have studied theoretically and experimentally. The manuscript presents the first experimental realization of such topological dislocations and will be of interest to a wide range of researchers. Overall, the paper is well organized in terms of theory and follows up with the corresponding experiment. All the theoretical claims were addressed and explained via reasonable experimental facts though it is lacking few details. But before recommending for publication, the following issues must be addressed: 1. It would be useful to clarify how this cavity mode is different from normal photonic crystal defect mode, e.g. in

term of robustness to dielectric fluctuation. In other words, how does this system compare with a topologically trivial system, at least in a simulation? See comment 5. ”

Our reply: We added investigations on the frequency stability and other differences from conventional photonic crystal defect modes in the Supplemental Materials. Upon our numerical simulations and experimental investigations, the topological cavity mode has the following differences between the conventional photonic crystal defect modes: First, the frequency of the topological cavity mode is more stable than the conventional defect mode. Using a normal dielectric photonic crystal of comparable band gap size, we show that the frequency of the conventional defect modes are more sensitive to perturbations (including (1) changing the radius of a nearby dielectric pillar and (2) changing the relative permittivity of a nearby pillar) than the topological cavity mode. Second, under the same perturbation (i.e., replacing a nearby dielectric pillar by a metallic pillar), the number of localized modes on a normal photonic crystal defect can be modified (i.e., one of the mode disappears), whereas there is always one topological cavity mode under such perturbations as confirmed by our simulations and experiments.

Reviewer: “2. Since the band gap is opening up between 3rd and 4th bands, the authors should specify the Chern number of 4th band so that the winding number of the edge state could be deduced.”

Our reply: The topological properties of the band gap are determined by all bands below the gap. In this sense, the Chern number of the 4th band does not affect the physics discussed in this work. Chern numbers of the first three bands are shown in Fig. 2 as red numbers. Specifically, the first two bands have zero Chern number, whereas the third band has Chern number 1. This is consistent with the winding number of the edge states (also shown in Fig. 2).

Reviewer: “3. Fig. 2c and d seem to be contradictory. The simulation in Fig. 2c shows the edge states spans from 12 to 12.5 GHz. But in experimental data of Fig. 2d the forward transmission is only present for the higher frequencies, around 12.5 GHz.”

Our reply: In Fig. 2d, pronounced nonreciprocal transmission appears in the frequency range of 12.21~12.84GHz. Relatively weak nonreciprocal transmission appears for lower frequencies as well. The bulk band gap is between 12.05 - 12.60 GHz. The edge states spectrum spans a much larger frequency range of 11-13.5GHz. Pronounced nonreciprocal transmission may appear only in a fraction of this range. This is possibly caused by the impedance mismatch between the feed probe (i.e., the antenna that launches the electromagnetic waves) and the edge/bulk states for the finite-size sample used in our experiments. We revised the manuscript to include the above discussions.

Reviewer: “4. Is there any explanation for the forward transmission yellow region in the 13-13.5 GHz region. In principle there should not be any transmission because of the bulk modes, unless the system size is large compared to localized bulk modes. This seems to be not the case.”

Our reply: The higher frequency window of 13-13.5 GHz is maybe associated with nonreciprocal edge states in the higher PBG (shown as shallow red and blue curves in Fig. 2c in the updated manuscript). These edge states are in common spectrum with the bulk states, yet the transmission seems to be dominated by the edge states. Such a phenomenon may be caused by impedance mismatch between the bulk states and the feed probe for the finite-size sample used in the experiments. However, since these edge states are in the higher PBG, they are not relevant to the aim of this work, i.e., to study the topological light trapping on the dislocation within the topological PBG.

Reviewer: “5. Why did the author only choose metallic defects and not just remove one of the YIG pillars? Is there any measure for the strength of the defect? How can one argue that something is a weak or strong perturbation, e.g. is it possible to compare the response to a topologically trivial system, as least in the simulation? In general, if one removes/perturbs a site in a photonic crystal the transmission is affected, but not completely vanished. 6. Have the authors measured/simulated the modification of the cavity mode frequency versus the defect strength? Intuitively, the transmission peak can move from fig-3-c to fig-3-d, while remaining in the bandgap.”

Our reply: To quantify the strength of the perturbation, we introduce two types of measure. In the first case, we modify the radius of a nearby dielectric pillar and use the relative change of the radius as a measure of the strength of the perturbation. In a second case, we modify the permittivity of a nearby dielectric pillar and use the relative change of the permittivity as a measure of the strength of the perturbation. In the revised manuscript, we added investigation on the frequency stability as functions of both measures and compare the results between the topological cavity mode and a topologically trivial photonic crystal defect mode. To ensure a fair comparison, the topologically trivial photonic crystal is chosen to be made of dielectric pillars of the same permittivity. The size of the trivial and topological PBG is the same, and the central frequency of the PBG is nearly the same. Our simulations are presented in the Supplemental Materials. The results indicate that the topological cavity mode is more stable in three different aspects: First, when the radius of a nearby pillar is changed, the frequency change of the topological cavity mode is considerably smaller than that of the trivial photonic crystal defect mode. Second, when the permittivity of a nearby pillar is altered, the same phenomena is observed. Third, when a nearby pillar is replaced with a metallic pillar, the number of localized modes for the conventional photonic crystal defect cavity changes from 2 to 1 (i.e., one of the localized mode disappears). In contrast, there is always one localized mode for the topological cavity when the same perturbations are applied.

REVIEWERS' COMMENTS:

Reviewer #1 (Remarks to the Author):

The authors have addressed all the concerns in my previous report. I am happy to recommend the publication of this work in Nature Communications.

Reviewer #2 (Remarks to the Author):

The reply letter and the revised manuscript adequately addressed the questions and comments. I recommend acceptance.

Reviewer #3 (Remarks to the Author):

second review

The authors have addressed most the concerns. Specifically, I would like to highlight two points from the previous round:
4) high transmission around 14GHz: I don't find authors response very convincing but I realized they have done their best to identify the origin of high peaks: (a) by recovering an extra pair of edge states (shaded ones in the band structure figure) and (2) impendence mismatch argument. If it's a bulk property, the peak should go down by increasing the system size. This can be seen in a simulation (this is an optional suggestion)

5) robustness: Indeed, the simulation Fig.S11 exhibits robustness. Although I find it difficult to call it "considerable". I am satisfied with the response.

I recommend the publication of the manuscript.

Ref#NCOMMS-18-05102A

Reviewer #3 (Remarks to the Author):

The authors have addressed most the concerns. Specifically, I would like to highlight two points from the previous round:
4) high transmission around 14GHz: I don't find authors response very convincing but I realized they have done their best to identify the origin of high peaks: (a) by recovering an extra pair of edge states (shaded ones in the band structure figure) and (2) impendence mismatch argument. If it's a bulk property, the peak should go down by increasing the system size. This can be seen in a simulation (this is an optional suggestion)
5) robustness: Indeed, the simulation Fig.S11 exhibits robustness. Although I find it difficult to call it "considerable". I am satisfied with the response.

I recommend the publication of the manuscript.

Our reply: We did the simulation for a sample with larger size ($39a_x$ along the x direction, $10a_y$ along the y direction). The transmission is obtained by a point-excitation and point-detection scheme along the boundary between topologically nontrivial and trivial photonic crystals. The setup and material parameters in the simulation are the same as those in the experimental measurements (of which the results are presented in Fig. 2d in the main text). The simulation results are presented in the figure below. The results show that: (I) perfect unidirectional transmission for the frequency window 12.1-12.7GHz (i.e., between the 3rd and the 4th bands), which can be associated to the chiral edge states; (II) The nonreciprocal photon transmission in the higher frequency window, about 13-13.5 GHz, is associated with both the bulk and edge modes. Observation (II) is supported by the fact that the backward transmission is also nonzero and pronounced in this higher frequency window. In contrast, for the lower frequency window, the backward transmission vanishes, which is in agreement with the bulk band gap and the chiral edge states. The above observations indeed agree with our band diagrams for the bulk and edge states in Figure 2c in the main text.

We improved the statements and the figure presentation in the Fig. S11 to indicate clearly that there is a pronounced difference between normal and topological cavity modes. Particularly, the number of modes for a normal cavity can be changed due to perturbations. This change is because some modes merge into (or emerge from) the bulk bands. In contrast, the emergence of the topological cavity mode is determined by the dual topology mechanism, which is robust to the perturbations. The frequency stability of the topological cavity mode is thus a reflection of the robustness of the topological cavity mode.

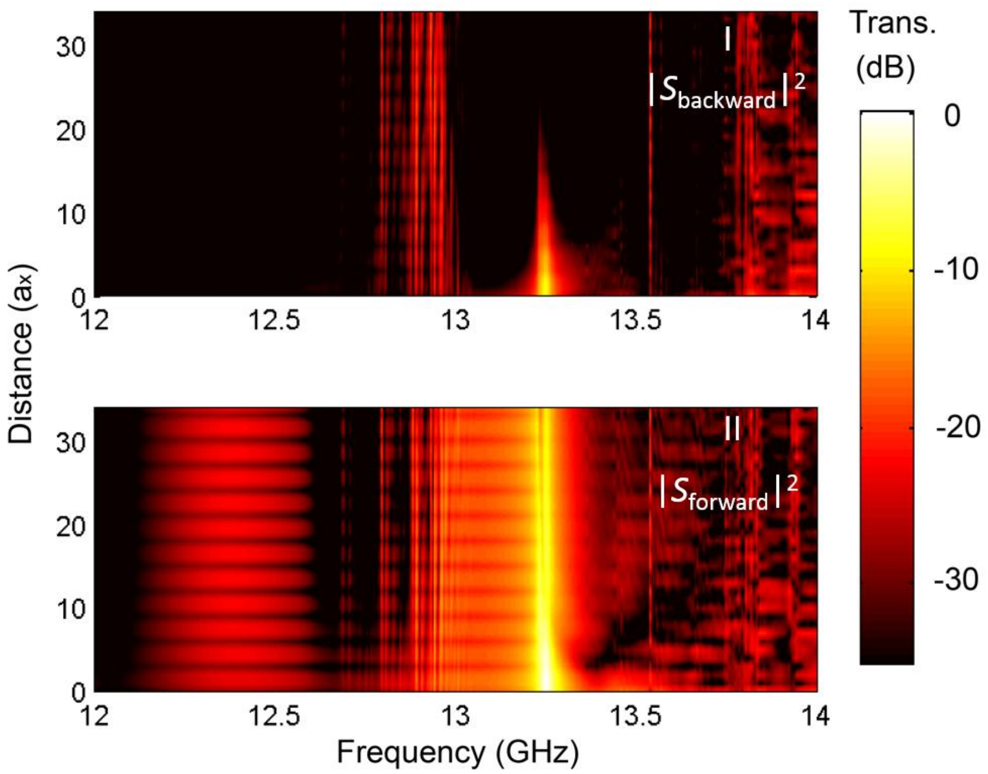

I
$|S_{\text{backward}}|^2$
II
$|S_{\text{forward}}|^2$
Distance (a_x)
Frequency (GHz)
Trans. (dB)
0
-10
-20
-30
12
12.5
13
13.5
14
0
10
20
30
